# Stagewise keratinocyte differentiation from human embryonic stem cells by defined signal transduction modulators

**DOI:** 10.7150/ijbs.44414

**Published:** 2020-02-21

**Authors:** Hui Zhong, Zhili Ren, Xiaoyan Wang, Kai Miao, Wenjun Ni, Ya Meng, Ligong Lu, Chunming Wang, Weiwei Liu, Chu-Xia Deng, Ren-He Xu, Guokai Chen

**Affiliations:** 1 Centre of Reproduction, Development and Aging, Faculty of Health Sciences, University of Macau, Taipa, Macau; 2 Cancer Centre, Faculty of Health Sciences, University of Macau, Taipa, Macau; 3 Department of Urology Surgery, Zhuhai People's Hospital, Jinan University, Zhuhai, Guangdong 519000, China; 4 Zhuhai Precision Medical Center, Zhuhai People's Hospital, Jinan University, Zhuhai, Guangdong 519000, China; 5 Center of Interventional radiology, Zhuhai People's Hospital, Jinan University, Zhuhai, Guangdong 519000, China; 6 State Key Laboratory of Quality Research in Chinese Medicine, Institute of Chinese Medical Sciences, University of Macau, Taipa, Macau; 7 Bioimaging and Stem Cell Core Facility, Faculty of Health Sciences, University of Macau, Taipa, Macau

**Keywords:** human embryonic stem cells, keratinocyte differentiation, defined factors

## Abstract

Keratinocyte is the predominant cell type in the epidermis of skin, and it provides the protective barrier function for the body. Various signaling pathways have been implicated in keratinocyte differentiation in animal models; However, their temporal regulation and interactions are still to be explored in pluripotent stem cell models. In this report, we use human embryonic stem cells to demonstrate that epidermal ectoderm and subsequent keratinocyte cell fate can be determined step by step under the regulation of defined factors. The inhibition of TGFβ initiates ectodermal lineage differentiation, and the activation of BMP pathway drives epidermal *TP63* expression. Meanwhile, the timely activation of WNT pathway suppresses extraembryonic lineage, and promotes epidermal cell fate. With further specification by NOTCH inhibition, more than 90% of cells become TP63-positive stage Ⅱ keratinocytes. Finally, stage Ⅲ keratinocytes are produced under defined hypo-calcium keratinocyte culture conditions, and are further matured in mouse xenograft model. This study not only establishes an *in vitro* platform to study keratinocyte cell fate determination, but also provides an efficient protocol to produce keratinocytes for disease models and clinical applications.

## Introduction

The skin separates organisms from the external environment and provides an essential protective barrier for the individual. The human skin consists of three layers: epidermis, dermis and hypodermis. The epidermis is the surface layer of the skin, and more than 90% of epidermal cells are keratinocytes 1, 2. Keratinocytes form a highly specialized epithelial layer to protect the body from infection and the loss of water. Keratinocytes generated from human pluripotent stem cells (hPSCs) are very useful in basic research, disease models and wound healing. However, few current procedures explore the details of keratinocyte cell fate determination *in vitro.*

The induction of epidermal cell fate normally requires a stagewise differentiation of epiblast cells specified by various signaling pathways, as revealed by studies in multiple animal models, including zebrafish, xenopus, chicken and mouse 3-6. During embryogenesis, epiblast cells are induced to ectoderm-like progenitors by TGFβ/Nodal/Activin inhibition 7, 8. Subsequently, BMP and WNT signals jointly induce the epidermal lineage 7, 9, 10. Meanwhile, the inhibition of NOTCH and FGF pathways promotes the epidermal fates 11, 12, 13. The temporal regulation of the involved pathways has been shown to be crucial for the cell fate determination among neural crest, neural plate border and epidermal cell fates 4, 11. Throughout epidermal differentiation, keratinocyte gene expression is tightly controlled and programmed. The *TP53* homolog *TP63* is the first transcription factor specific for the epidermal lineage 14. TP63 is essential for epidermal proliferation and stratification. It encodes two isoforms: △Np63, expressed in the basal layer, and TAp63, which is more highly expressed in suprabasal cells 13. TP63 subsequently induces the expression of key keratinocyte genes, such as KRT14, KRT1 and KRT10, during keratinocyte differentiation 6.

Human embryonic stem cells (hESCs) have the potential to generate all cell types in our body, and they provide an ideal model system to study human embryogenesis 15. To generate keratinocytes from hESCs, people have used serum, retinoic acid or dual inhibition of TGFβ/BMP pathways to initiate ectoderm differentiation 16-18; BMP4, FGF inhibitor or NOTCH inhibitor is then used in different procedures to drive epidermal cell fate 13, 18, 19. In the end, low-calcium keratinocyte medium is always used to enrich and expand the derived keratinocytes. Most procedures use undefined conditions, which makes it difficult for researchers to appreciate the details of molecular regulation in the process.

Although all the current procedures could generate keratinocytes, there are still major issues to be addressed. Firstly, it is unclear how keratinocyte cell fate is determined stage by stage *in vitro*. Current studies focus on the analysis after the enrichment by keratinocyte media, and it is unknown whether the differentiation procedures are efficient to generate high purity, P63-positive keratinocyte before the enrichment. Secondly, the induction procedures often do not reflect the findings from animal models 14, 16, 20-24. For instance, WNT is essential for epidermis development in animal models; However, there is no report on how WNT could affect hESC differentiation toward keratinocytes 2, 4. Thirdly, there is no study to explore the interaction of multiple key pathways in keratinocyte induction from hESCs.

As discussed above, TGFβ, BMP, WNT and NOTCH pathways are essential for *in vivo* keratinocyte differentiation, but these pathways have also been implied in the differentiation of multiple other lineages (Figure S1A). For example, TGFβ inhibition, BMP activation and NOTCH inhibition can all induce extraembryonic lineage 25, 26, while WNT signaling drives both neural crest and mesodermal differentiation 27, 28. In order to promote stage Ⅲ keratinocyte differentiation, it is important to suppress the alternative cell fates that could be induced by these signals. We hypothesize that temporal and combinatory regulations of the key pathways are critical to efficiently induce epidermal and keratinocyte cell fates while suppressing differentiation toward neural, extraembryonic and other lineages. We try to develop a defined differentiation procedure in a stagewise manner based on knowledge of *in vivo* studies. In this report, we examined the roles of key signaling pathways at each stage of epidermal differentiation and established a keratinocyte differentiation procedure under defined conditions (Figure S1B). We demonstrated that TGFβ inhibition initiated ectoderm differentiation, and dual activation of BMP and WNT pathways drove epidermal specification. We also showed that NOTCH inhibition and hypo-calcium conditions promoted further keratinocyte maturation. Through stepwise modulation of specific pathways, we were able to effectively generate stage Ⅱ and stage Ⅲ keratinocytes from hESCs and human induced pluripotent stem cells (hiPSCs). This study provides a novel research platform for people to study epidermal differentiation and to develop related applications.

## Materials and Methods

### Human ESC culture

Human ESCs (H1 and H9) and iPSCs (ND1-4, NL-1, NL-4) were used in this study. H1 was the main cell line used in keratinocyte differentiation study, and the keratinocyte differentiation protocol was confirmed by H9, ND1-4, NL-1, and NL-4. All the cell lines were maintained in E8 medium (Chen et al., 2011) on Matrigel-coated plates (Corning 354230). Cells were passaged every 3-4 days using EDTA method (Liu and Chen, 2014) in the presence of ROCK inhibitor (Y27632, 5µM). The ROCK inhibitor was removed the next day, and the medium changed daily.

### Keratinocyte differentiation in monolayer

Human PSCs were cultured as above until 30% confluence, then switched to differentiation medium (DMEM/F12, L-ascorbic acid, selenium, transferrin, insulin, 1x chemically defined lipid concentrate. From Day 0 to Day 8, cells were cultured in differentiation medium with the following treatments: Day 0-6, 10 µM SB431542; Day 1-6, 5 µM CHIR99021; Day 1-8, 10 ng/ml BMP4 (R&D 314-BP-01M); Day 4-8, 5 µM DAPT (Tocris 2634). From Day 9 to Day 11, the medium was changed to low-calcium differentiation medium (containing 0.06 mM calcium) supplemented with BMP4/DAPT/EGF (10 ng/ml). The resulting stage Ⅲ keratinocyte were then maintained in low-calcium differentiation medium supplemented with 0.7% BSA (Sigma, A7030), 2.4 ng/ml adenine (Sigma, A8626-100G), 0.4 µg/ml hydrocortisone (Sigma, H0888-1G), 5 µg/ml NKH477 (Tocris, 1603), 10 ng/ml EGF (Rasmussen et al., 2013). Components of the low-calcium differentiation medium: DMEM/F12 (-Calcium) (Sigma, D9785), 28.64 mg/L magnesium chloride (Sigma, M4880), 28.84 mg/L magnesium sulfate (Sigma, M2643), 1200 mg/L sodium bicarbonate (Sigma, S5761), 7.5 mg/L L-Glutamine (Sigma, G5792), 59.05 mg/L L-Leucine (Sigma, L8912), 91.25 mg/L L-Lysine monohydrochloride (Sigma, L8662), 17.24 mg/L L-Methionine (Sigma, M5308), and calcium chloride (Sigma, C5670) supplemented at designated concentrations. Working concentrations of treatments tested but not included in the final procedure: FGF2, 20 ng/ml; PD0325901, 25 nM; SU-5402, 10 μM (TOCRIS, 3300). The concentrations of chemicals and growth factors used in all experiments are the same as above unless otherwise specified.

### Real Time Polymerase Chain Reaction

RNA was purified using RNAiso Plus reagent (Takara #9109) according to manufacturer's protocol. Reverse transcription was carried out using High-Capacity cDNA Reverse Transcription Kit from Applied Biosystems (4368814). Real‐time PCR was performed using Takara SYBR® Premix Ex Taq™ II on Applied Biosystems QuantStudio 7 Flex Real-time PCR System and the data were normalized to GAPDH or TBP expression. Primers used were listed in supplemental table S2.

### Immunostaining

Cells were fixed in 4% paraformaldehyde for 20 min, washed with PBS, and permeabilized in 0.5% Triton X-100/PBS for 10 minutes. After blocking with 3% BSA/PBS for 1h, cells were incubated with primary antibodies overnight in 4℃. Cells were then washed in PBS, incubated with the secondary antibodies for 1 hour and washed with PBS three times. Hoechst 33258 (Molecular Probes H1398) was used for nuclei staining (10min). The following primary antibodies were used: anti-TP63 antibody (BA1887, Boster); Cytokeratin 14 (sc-58724, Santa Cruz); Cytokeratin 1 (sc-65999, Santa Cruz); Cytokeratin 10 (sc-23877, Santa Cruz); Involucrin (sc-21748, Santa Cruz); Filaggrin (sc-30229, Santa Cruz); KRT18 (sc-6259, Santa Cruz).

### Flow Cytometry Analysis

Cells were dissociated into single cells by TrypLE Select enzyme (Thermo Fisher Scientific) treatment, washed in PBS, and then fixed by 4% paraformaldehyde (PFA) for 20 minutes at room temperature. Fixed cells were washed with PBS and permeabilized in 0.5% Triton X-100/PBS for 10 minutes. After washes in PBS, cells were blocked in 3 % bovine serum albumin (BSA/PBS) for 1 hour and incubated with primary antibodies overnight in 4℃. After washing in PBS for 3x 5 minutes, cells were incubated with secondary antibodies for 30 minutes at room temperature, washed twice, and suspended in PBS for flow cytometry analysis using BD Accuri™ C6 Cytometer. Primary antibodies used: Cytokeratin 14; Cytokeratin 1.

### IncuCyte Zoom Live-Cell Imaging

Cell proliferation was measured with the IncuCyte Zoom Live-Cell Imaging System (Essen Bioscience). Cells were seeded in a Matrigel coated 6-well plate at 10^5^ cells per well, and nine images were captured in each well every 2 hours over a 4-day period. Proliferation was determined by calculating the total area occupied by cells (% confluence), and the starting confluence was around 16%.

### Microarray analysis

Total RNA was extracted from collected cell samples (RNAiso Plus reagent, Takara #9109) and further purified (RNAeasy mini kit, QIAGEN). cRNA was produced using the TargetAmpTM-Nano Labeling Kit. cRNA samples were hybridized onto microarrays using the HumanHT-12 v4 Expression BeadChip Kit (Illumina) and the arrays were scanned on an iScanner (Illumina).

The microarray data was pre-processed via the arrayanalysis.org webportal (www.arrayanalysis.org) and the on-line Illumina pre-processing pipeline was used. Briefly, box plot and PCA plot were used to inspect the data quality. Background correction and quantile normalization were applied to the raw data. Then the log2 transformation was employed as the variance stabilizing strategy. Heatmaps were drawn with the R package (pheatmap) to show the expression patterns. For the global gene expression, hierarchical clustering was applied to both axes using euclidean metric for similarity and complete linkage clustering for row clustering and single linkage clustering for column clustering. The GEO accession number for the microarrays data reported in this paper is GSE144241.

### Mouse excisional wound model and keratinocyte transplantation

All the animal experiments were conducted under an animal use protocol approved by the University of Macau Sub-panel on Animal Research Ethics. NOD SCID mice (6-8 weeks old; male; body weight 20-23 g; n=10) were obtained from the animal facility of University of Macau.

After anesthesia, hair was removed from the dorsal surface of NOD SCID mice, and an 8-mm punch was used to mark the edge of wounded skin. The full thickness skin flap was elevated with scissors. A GFP-labeled H1 cell line was generated by constitutively expressing a GFP construct at the AAVS1 site (Mali et al., 2013; Qian et al., 2014), (donor plasmid AAVS1-CAG-hrGFP, Plasmid#52344, Addgene; Cas9 plasmid px330-U6-Chimeric_BB-CBh-hSpCas9, Plasmid#42230). The GFP-labeled cells were then differentiated to keratinocytes according to our differentiation protocol. After 20 days of differentiation, 0.7x106 cells were mixed with matrigel and injected into the skin flap. Additional 0.3x106 cells were mixed with matrigel and topically applied onto the wound bed. After transplantation, the skin flap was returned and sutured to prevent cell loss. Seven days after the surgery, the animals were sacrificed, and the skin was removed and perfused with 4% paraformaldehyde for immunostaining and H&E staining.

### 3D organotypic culture

Three-dimensional culture of hESC-derived keratinocytes were carried out according to published procedures 29. Briefly, human foreskin fibroblasts were embedded in a collagen matrix and cultured on 3.0µm polycarbonate membrane inserts for 7 days to form dermal equivalents. Keratinocytes derived from hESCs were collected on day 20 of differentiation and seeded on the collagen lattice. Air-lift was performed after one week, and the keratinocytes were further cultured for two weeks at the air-liquid interface before collection for immunohistochemistry analysis.

### Statistical analysis

All data are presented as the mean± SD of three or more independent experiments unless otherwise specified.

Statistical significance was determined by two-tailed Student's t-test. P < 0.05 was considered statistically significant.

## Results

### Primitive ectoderm cell fate determination through TGFβ inhibition

**Figure 1:**
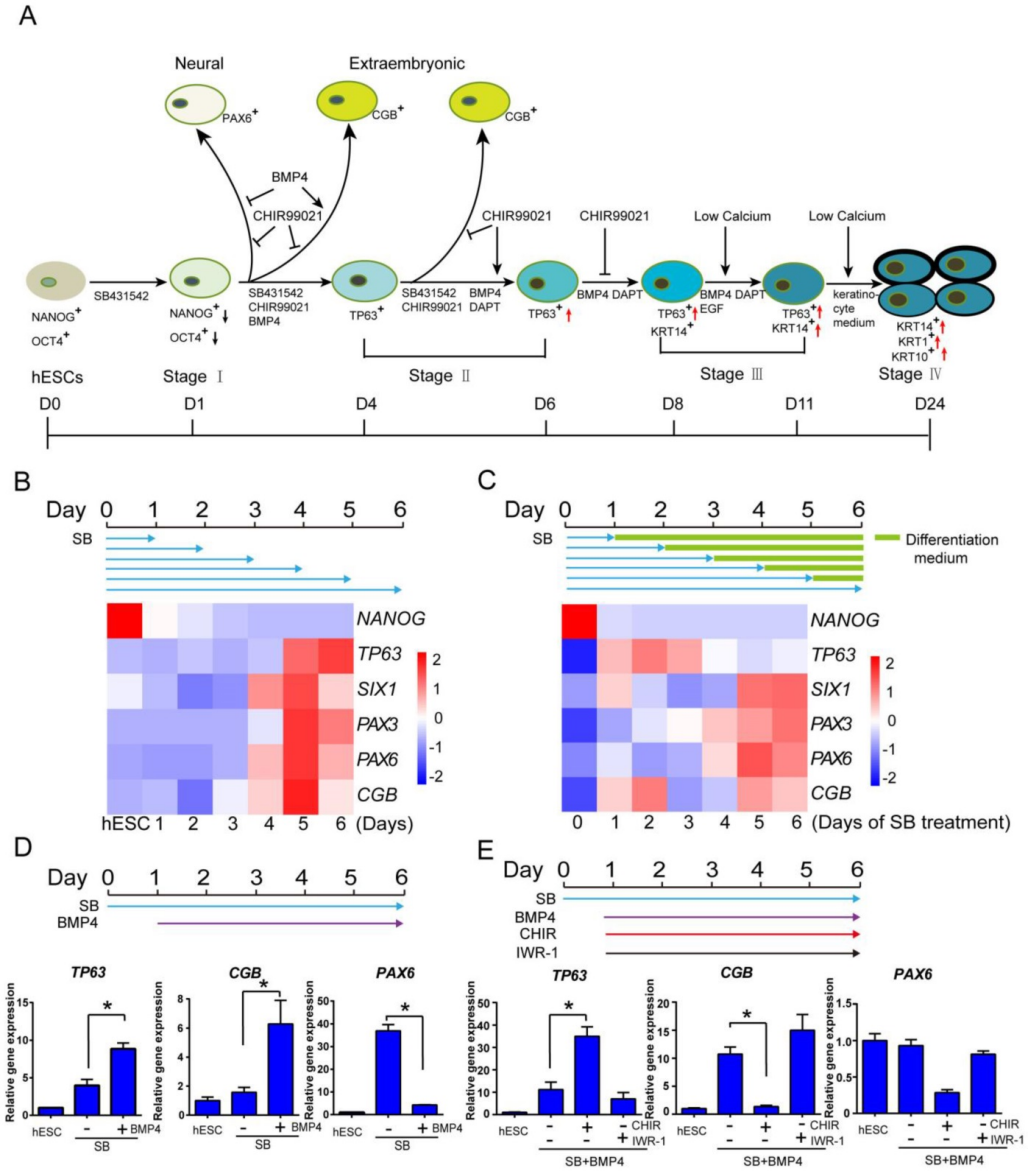
** Early epidermal cell fate determination under the influence of multiple signaling pathways in hESCs. See also Figure S1. A.** Stagewise method for keratinocyte differentiation. **B.** The time course of epidermal differentiation under TGFβ inhibition. hESCs were treated with SB431542 (SB) in differentiation medium (See Materials and Methods), and the cells were harvested at specific time points for analysis of gene expression by RT-qPCR. The results were normalized to GAPDH. **C.** The emergence of *TP63* under pulse treatment of TGFβ inhibitor. hESCs were treated with SB431542 (SB) for specified periods in differentiation medium followed by differentiation medium only until day 6. Cells were collected on day 6 for analysis of gene expression by RT-qPCR. The results were normalized to GAPDH. **D.** BMP4 promotes TP63 expression under TGF-β inhibition. hESCs were treated with SB431542 (SB) in differentiation medium, and BMP4 was added from day 1 till day 6 when cells were harvested for gene expression analysis by RT-qPCR. The results were normalized to hESC; Data are presented as the mean±SD of three independent experiments. *, p < 0.05. **E.** The impact of WNT pathway modulation on epidermal cell fate determination. hESCs were treated with SB431542 (D0-6) and BMP4 (D1-6) in differentiation medium, and CHIR99021(CHIR, 5 µM) or IWR-1 (1 μM) was added from day 1 to day 6. Cells were then harvested for gene expression analysis by RT-qPCR. The results were normalized to hESC; Data are presented as the mean±SD of three independent experiments. *, p < 0.05. ns, not significant.

In order to generate keratinocytes for disease models and potential therapeutic applications, we tried to establish an *in vitro* keratinocyte differentiation platform in defined medium. Based on the knowledge from animal models, we systematically analyzed the impact of key pathways at each developmental stage in keratinocyte cell fate determination. We identified TGFβ, BMP4, WNT, NOTCH and calcium level as the main players in the differentiation, and they need to be temporally modulated to achieve optimal keratinocyte derivation (Figure 1A). The details of protocol development are elaborated below.

First, we examined whether TGFβ inhibition could induce primitive ectoderm from hESCs, which has the potential to become neuroectoderm and surface ectoderm. ALK5 inhibitor SB431542 was reported to inhibit TGFβ/Activin/Nodal pathway in ectodermal initiation in mouse model 7. We tested this treatment in a 6-day time course, and examined the emergence of lineage-specific marker genes from day 1 to day 6. Under TGFβ inhibition, the pluripotency marker *NANOG* decreased significantly in one day, while all ectodermal and extraembryonic makers emerged in six days (*TP63*, epidermis marker; *PAX3*, neural crest; *SIX1*, placode; *PAX6*, neural; *CGB* gene family, extraembryonic) (Figure 1B). It suggests that TGFβ inhibition initiates differentiation and induces all ectodermal lineages, and a 6-day time frame could be used to evaluate the emergence of surface ectoderm and epidermal lineages.

**Figure 2:**
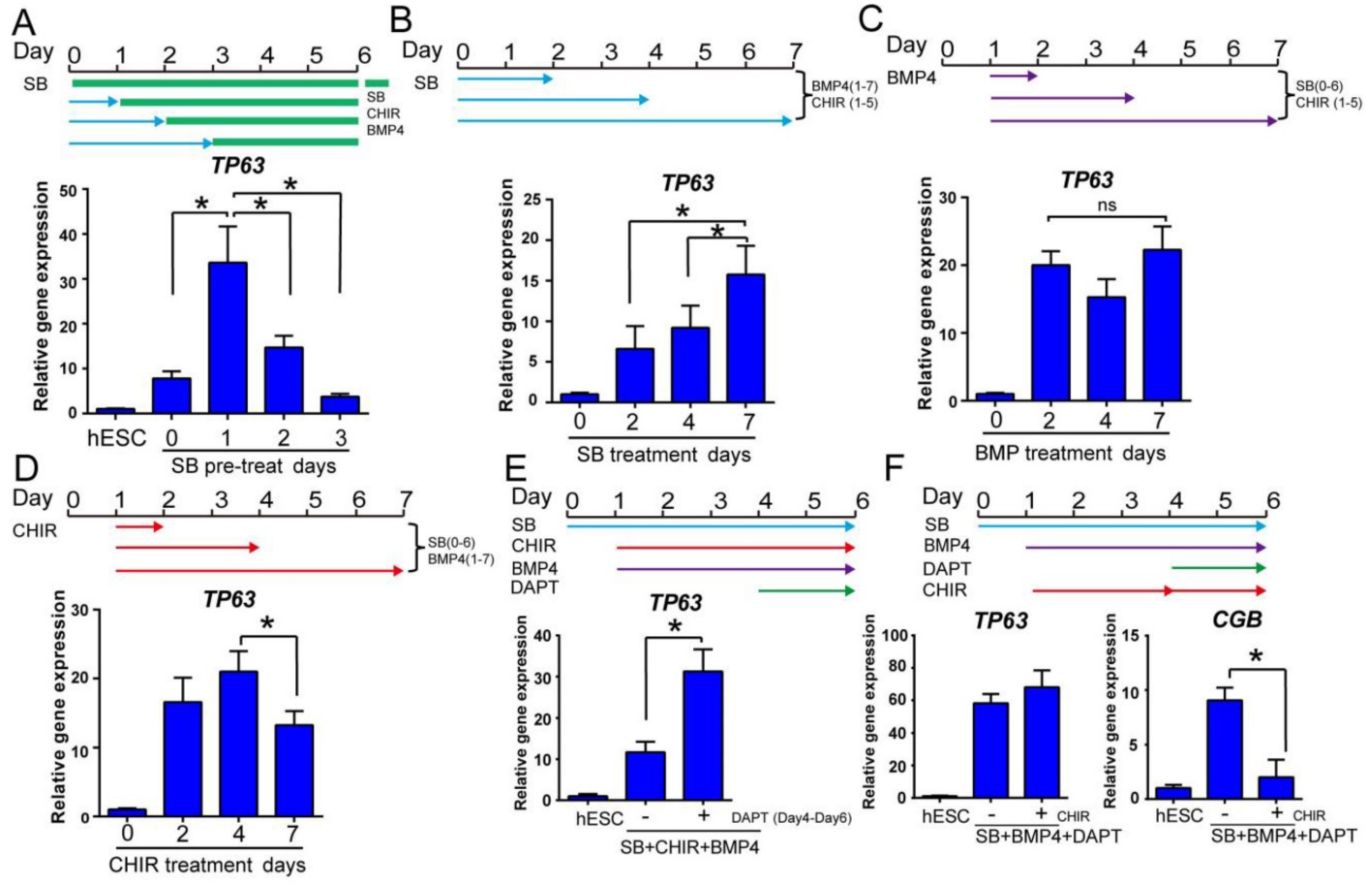
** Optimization of epidermal cell fate determination at early stage. See also Figure S2. A.** The time course of primitive ectoderm induction. Cells were treated with SB431542 single treatment for 0, 1, 2 or 3 days in differentiation medium, followed by combination treatment with SB431542, BMP4 and CHIR99021 until day 6, and collected on day 6 for *TP63* gene expression analysis by RT-qPCR. The results were normalized to hESC; Data are presented as the mean±SD of three independent experiments. *, p < 0.05. **B.** The impact of prolonged exposure to TGFβ inhibitor. H1 cells were treated with TGFβ inhibitor SB431542 (SB) for 2, 4 or 7 days in the presence of BMP4 (Day 1 to 7) and CHIR99021 (CHIR, Day 1 to 5) in differentiation medium, and gene expression was analyzed on day 7 by RT-qPCR. The results were normalized to control (no SB4315432 treatment); Data are presented as the mean±SD of three independent experiments. *, p < 0.05. **C.** The effect of BMP4 timing on *TP63* induction. In the presence of SB431542 (SB, Day 0-6) and CHIR99021 (CHIR, Day 1-5), BMP4 was added for specific time periods, and gene expression was analyzed on day 7 by RT-qPCR. The results were normalized to control (no BMP4 treatment); Data are presented as the mean±SD of three independent experiments. ns, not significant (one-way ANOVA with post hoc multiple comparisons). **D.** The effect of WNT activation timing on epidermal differentiation. WNT activator CHIR99021 (CHIR) was added in the presence of SB431542 (SB, Day 0-6) and BMP4 (Day 1-7) for specified periods of time, and gene expression was analyzed by RT-qPCR on day 7. The results were normalized to control (no CHIR treatment); Data are presented as the mean±SD of three independent experiments. *, p < 0.05. **E.** Impact of NOTCH inhibition during epidermal differentiation. Epidermal differentiation was carried out with SB431542 (SB, Day 0-6), CHIR99021 (CHIR, Day 1-6) and BMP4 (Day 1-6). NOTCH inhibitor DAPT was added between Day 4-6, and the gene expression was analyzed by qPCR on Day 6. The results were normalized to hESC; Data are presented as the mean±SD of three independent experiments. *, p < 0.05. **F.** The impact of WNT activation along with NOTCH inhibition on epidermal cell fate determination. Cells in epidermal differentiation were treated with DAPT with or without CHIR99021 (CHIR) between day 4 to 6, and the samples were collected on Day 6 for RT-qPCR. The results were normalized to hESC; Data are presented as the mean±SD of three independent experiments. *, p < 0.05.

We then evaluated whether the duration of TGFβ inhibition could affect ectodermal differentiation in a set of pulse-release treatments. hESCs were first exposed to TGFβ inhibition for different periods of time, and then allowed to differentiate without the inhibition till day 6. We found that one day of TGFβ inhibition was sufficient for the emergence of epidermal, placode and extraembryonic lineages, but neural and neural crest lineage markers required more than five days of TGFβ inhibition (Figure 1C). We also noticed that the extended TGFβ inhibition by itself relatively suppressed epidermal lineage, while increasing the differentiation toward other lineages (Figure 1C). It suggests that primitive ectoderm differentiation is triggered after one day of TGFβ inhibition, and additional modulation is probably needed to boost epidermal differentiation and suppress other lineages. Based on other data discussed later in the manuscript (Figure 2), we chose to use 6-day SB treatment to optimize surface ectodermal differentiation, and various treatments were applied to cells on day one or later.

### Epidermal specification through BMP and WNT activation

BMP4 is widely used in epidermal differentiation, and we tested its effect in combination with continuous TGFβ inhibition. BMP4 significantly enhanced the expression of epidermal marker *TP63* and suppressed neural lineage marker *PAX6*, while elevating the expression of extraembryonic gene *CGB* (Figure 1D). This result suggests that BMP4 along with SB431542 drove not only epidermal but also extraembryonic differentiation. In order to generate epidermal specific lineage, additional factor is needed to suppress the extraembryonic lineage.

In order to identify the factors that could suppress extraembryonic lineage, we screened various pathway modulators in the presence of BMP4 and SB431542. We found that WNT activator CHIR99021 significantly inhibited extraembryonic marker *CGB*, while increasing epidermal marker *TP63* expression (Figure 1E). In contrast, WNT inhibitor IWR-1 suppressed epidermal lineage and increased extraembryonic differentiation (Figure 1E). CHIR99021 alone or along with BMP4 further suppressed the expression of neural marker *PAX6*, and CHIR99021 alone inhibits *TP63* expression when BMP4 was not added (Figure S1C and S1E). These data indicate that WNT activation is important for epidermal lineage differentiation, which is consistent with the findings in animal models 4, 11.We also checked the effect of FGF/MEK/ERK signaling pathway modulators on keratinocyte differentiation, including MEK/ERK inhibitor PD0325901, FGF2 and FGF receptor antagonist SU-5402. These treatments showed some beneficial effects under specific settings, but not as strong compared to BMP4 or CHIR99021 in *TP63* induction (Figure S1D-E).

### Optimization of temporal treatments in early epidermal differentiation

The temporal regulation by growth factors is critical for efficient cell fate determination 30. We optimized the temporal treatments for epidermal ectoderm differentiation by SB431542, BMP4 and CHIR99021. In a 6-day time course, the best *TP63* expression was observed with one day of SB431542 single treatment before tri-reagent treatment with SB431542, BMP4 and CHIR99021 (Figure 2A). Although prolonged treatment with SB431542 alone suppressed epidermal lineage (Figure 1B), we found that extended SB431542 exposure was beneficial to *TP63* expression in the presence of BMP4 and CHIR99021 (Figure 2B). These findings suggest that early TGFβ inhibition along with CHIR99021 and BMP4 directs the differentiation toward epidermal ectoderm lineage.

Besides the temporal regulation of TGFβ, the duration of BMP4 and WNT activation are also important for *TP63* expression. In the presence of SB431542 and CHIR99021, extended BMP4 exposure led to consistent *TP63* expression (Figure 2C). Higher dose of BMP4 was also beneficial to *TP63* expression (Figure S2A). We also found that *TP63* expression was decreased by extended WNT activation in the presence of SB431542 and BMP4 (Figure 2D). When the dose of WNT activator was decreased, *TP63* was further elevated at the expense of increased extraembryonic marker CGB expression (Figure S2B). These results indicate that BMP and WNT signaling promote epidermal differentiation, but additional modulators may be necessary to overcome the WNT effect to drive neural crest cell fate^31^.

### NOTCH inhibition improves stage II keratinocyte differentiation

NOTCH inhibitor DAPT is reported to increase *TP63* expression in epidermal differentiation 13, and it is also an inducer for extraembryonic lineage 26. We demonstrated that *TP63* expression was increased by DAPT treatment from day 4 to day 6 (Figure 2E). Continued WNT activation was required at this stage, as DAPT treatment without CHIR99021 led to increase of both *TP63* and *CGB* expression without affecting neural and neural crest markers (Figure S2C). Adding CHIR99021 from day 4 to 6 helped suppress *CGB* and *PAX6* while maintaining *TP63* expression (Figure 2F and S2D). These results suggest that NOTCH inhibition is beneficial for the transition from primitive ectoderm to stage Ⅱ keratinocytes, but concomitant WNT activity is necessary to suppress extraembryonic and neural cell fates.

### Optimization of stage III keratinocyte differentiation

**Figure 3:**
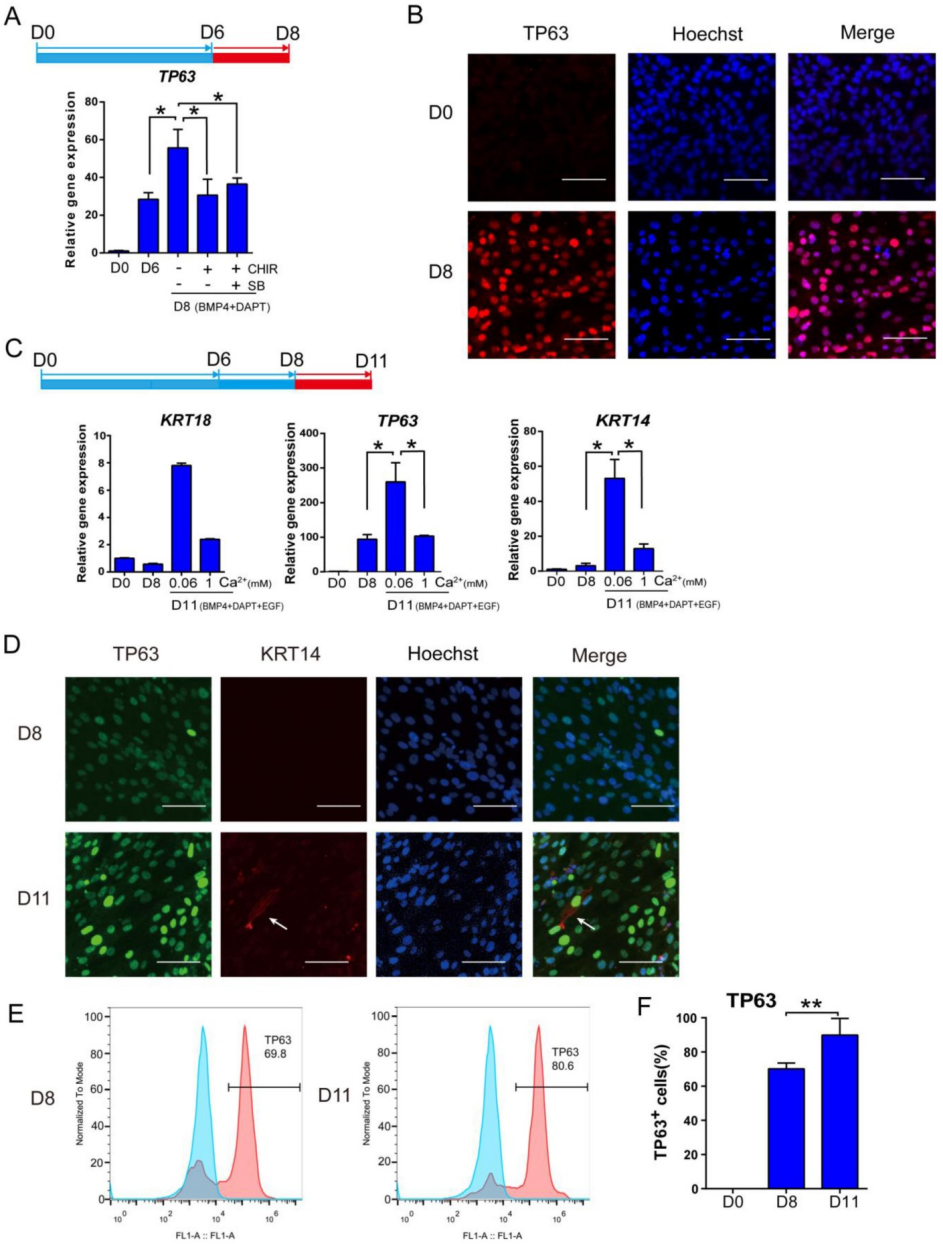
** Optimization of keratinocyte maturation conditions. See also Figure S3. A.** BMP4 and NOTCH inhibitor (DAPT) treatments are sufficient to support keratinocyte maturation from day 6 to day 8. Beyond Day 6 of differentiation, cells were treated with or without SB431542 (SB) or CHIR99021 (CHIR) under BMP4 and NOTCH inhibitor treatment for two extra days, and the gene expression was examined by RT-qPCR. The results were normalized to hESC; Data are presented as the mean±SD of three independent experiments. *, p < 0.05. **B.** Immunostaining of TP63 expression on day 0 and day 8. Scale bar, 50 µm. **C.** The impact of calcium concentration on the expression of keratinocyte markers. After eight days of differentiation, cells were maintained in different calcium concentrations (1 mM versus 0.06 mM) between day 9 and day 11, and analyzed for gene expression. The results were normalized to hESC; Data are presented as the mean±SD of three independent experiments. *, p < 0.05. **D.** Immunostaining of TP63 (green) and KRT14 (red) expression on D8 and D11. Scale bar, 50 µm. White arrow, KRT14 expression. **E.** Flow cytometry analysis of TP63 on Day 8 and Day 11 of differentiation. Blue peak represents undifferentiated hESCs; pink peak represents keratinocyte. **F.** Quantification of TP63-positive cells on Day 0, Day 8 and Day 11 by flow cytometric analysis. Data shown are mean±SD of three independent experiments. *, p < 0.05.

After the first 6 days of differentiation, additional treatment is necessary to promote the transition to stage Ⅲ keratinocytes. Continuous exposure to BMP4 and DAPT further increased *TP63* expression (Figure 3A). Addition of CHIR99021 or a combination of SB431542 and CHIR99021 was not beneficial to *TP63* expression between day 6 and day 8 (Figure 3A and S3A). Based on these observations, BMP4 and DAPT were used to drive keratinocyte maturation after day 6 without further CHIR99021 or SB431542 treatment. Immunostaining showed that more than 70% of cells were TP63-positive after 8 days of differentiation (Figure 3B and S3F).

Even though *TP63* was highly expressed at day 8, key keratinocyte marker *KRT14* level was still low by day 8 (Figure S3A). Epidermal growth factor (EGF) is a common factor in keratinocyte media 32, and we included EGF after day 8 in our protocol.

Low calcium concentration was reported to enhance keratinocyte differentiation 33, 34. We examined the effect of calcium on keratinocyte differentiation after day 8, because premature exposure led to inconsistent cell survival (data not shown). We observed that decreasing calcium to 0.06mM significantly increased *TP63* and *KRT14* expression (Figure 3C and S3B). We further evaluated the keratinocyte differentiation by immunostaining and FACS analysis. More than 80% of cells were TP63 positive, and KRT14 expression became detectable on day 11. The expression of TP63 and KRT14 were visibly higher in day 11 cells than those on day 8, which is consistent with the FACS results (Figure 3D-F and Figure S3D). E-cadherin, an essential regulator of keratinocyte organization 35, was also detectable at the periphery of cells on D8 (Figure S3C). These data suggest that stage Ⅲ keratinocytes were derived, but further maturation was necessary.

### Keratinocyte derivation in defined conditions

**Figure 4:**
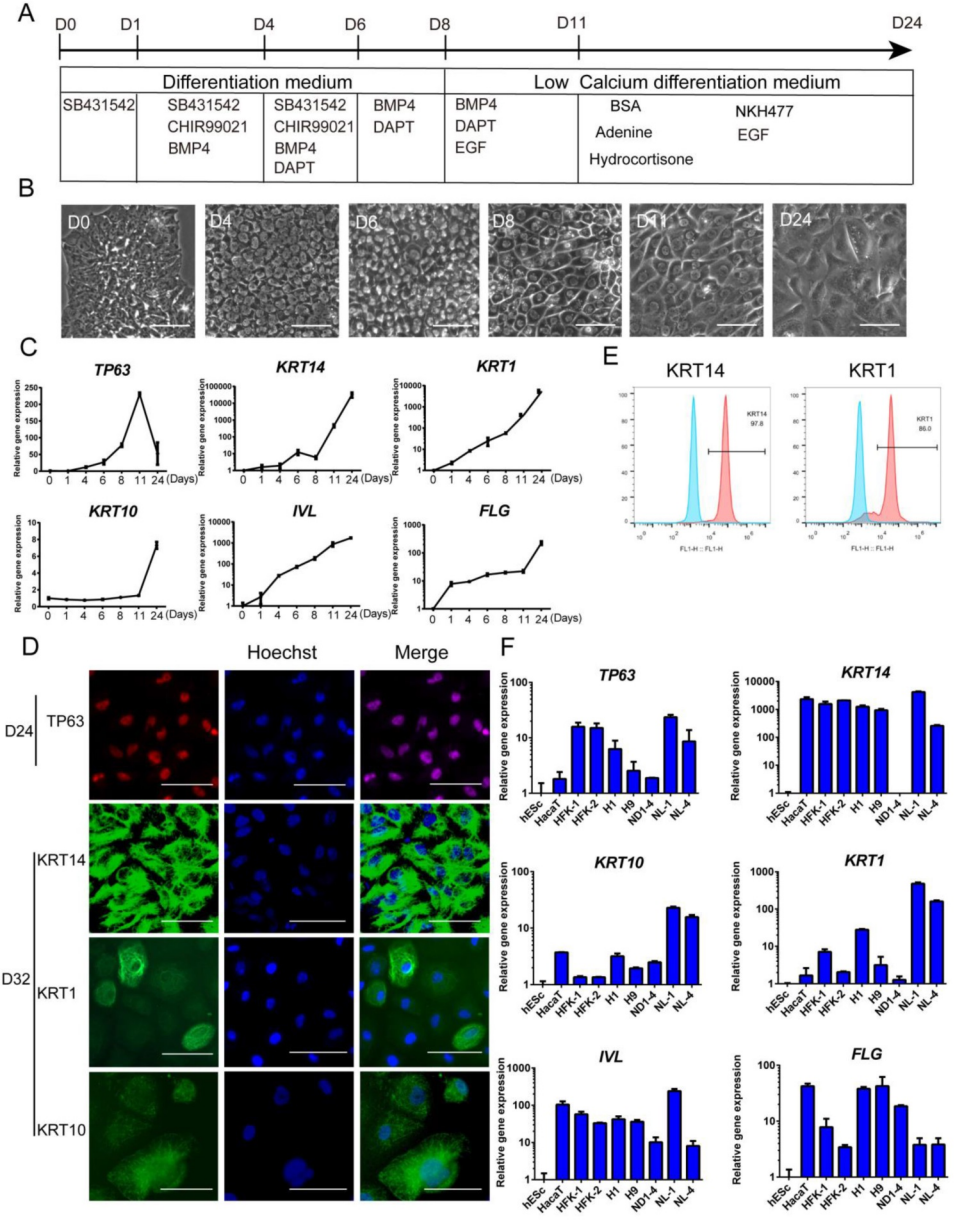
** Keratinocyte derivation procedure in defined conditions. See also Figure S4. A.** Schematic drawing of the optimized keratinocyte differentiation protocol. **B.** Cell morphology changes in the differentiation process. Scale bar,100µm. **C.** Stagewise keratinocyte gene expression by RT-qPCR. The results were normalized to hESC (D0); Data are representative of 3 independent experiments. **D.** Immunostaining of TP63 (red), KRT14 (green), KRT1 (green) and KRT10 (green) on day 24 and day 32 of differentiation. Scale bar, 50µm. **E.** Flow cytometry analysis of KRT14 (left panel) and KRT1 (right panel) on Day 28 of differentiation. Blue peak represents undifferentiated hESCs; pink peak represents keratinocyte. **F.** Keratinocyte differentiation from multiple hESC (H1, H9) and hiPSC (ND1-4, NL-1, NL-4) lines. Gene expression was analyzed on day 20 of differentiation and compared with HaCaT keratinocyte cell line, primary human foreskin keratinocytes (HFK-1, HFK-2) and undifferentiated hESCs (H1). The results were normalized to hESC (H1); Data are representative of 3 independent experiments.

Based on the above findings, we were able to differentiate hESCs to keratinocyte lineage, and we then cultured the cells further in a defined medium that was formulated according to previous reports by others 36. Finally, we established a procedure to differentiate hESCs toward keratinocytes through multiple stages (Figure 4A). In the process, we observed significant changes in the cellular morphology (Figure 4B). The epidermal marker gene expression changes were consistent with differentiation stages (Figure 4C). After the cells were differentiated for more than 20 days, most cells expressed keratinocyte markers, including TP63, KRT14, KRT10 and KRT1 (Figure 4D and S4D). This observation was consistent with flow cytometry results that >95% of the cells were positive for KRT14 and > 85% of cells express maturation marker KRT1 (Figure 4E). This differentiation process can also be carried out in fully chemically defined culture conditions, in the absence of Matrigel and albumin. We showed that keratinocytes were generated on recombinant vitronectin-coated surface (Figure S4A), and in albumin-free conditions (Figure S4B). The keratinocytes generated have high expansion capacity (Figure S4C) and can be passaged 8-10 times *in vitro*.

The keratinocyte differentiation procedure was then applied to multiple hPSC lines, including hESC (H1, H9) and hiPSC (NL-1, NL-4, ND1-4) lines. Most cell lines showed positive expression of keratinocyte genes, and the levels were comparable to the immortalized keratinocyte cell line HaCaT and primary human keratinocyte cell lines (Figure 4F). We also noticed that different cell lines vary in the level of marker gene expression. It indicates that the protocol probably needs to be tweaked to fit the differentiation needs of specific cell lines.

### Gene Expression in Keratinocyte Differentiation

**Figure 5:**
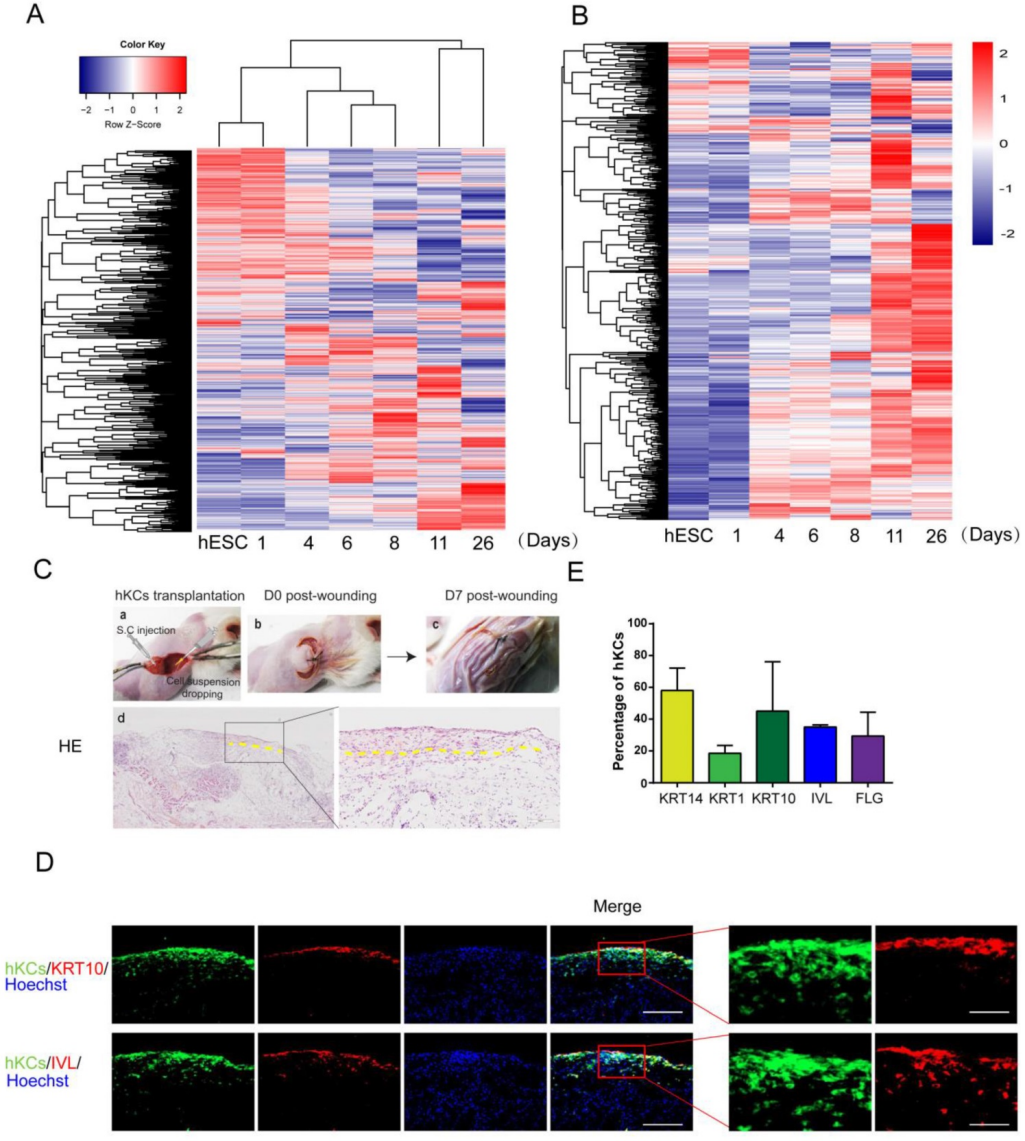
** Further analysis of derived keratinocytes by global gene expression and transplantation. A.** Cluster of global gene expression profiles during keratinocyte differentiation. RNA samples are collected at each stage of keratinocyte differentiation, and gene expression was analyzed by microarray. The global gene expression is clustered by Complete-linkage clustering method. **B.** Expression of epidermal-specific genes through the differentiation process. Epidermal specific genes were selected according to published dataset, and the pattern of gene expression was displayed by stage of differentiation. **C.** hESC-derived keratinocyte (hKC) transplantation in mouse excisional wound model. a. Representative image of mouse excisional wound and sites of hKC application. b. Representative image of the wound right after transplantation. Exposed wound areas were marked with yellow dash line. c. Representative image of the wound seven days after wounding and cell transplantation. The skin area was processed for further study. d. H&E staining of the wound healing site seven days after keratinocyte transplantation. Square marks the area shown as high magnification image on the right. Scale bar, 400µm on the left; 100µm on the right. **D.** Immunostaining of KRT14, KRT1, KRT10, IVL and FLG (red fluorescence) on serial skin sections. Samples were collected seven days after transplantation of keratinocytes derived from GFP-labeled hESCs (green fluorescence). Blue fluorescence, nuclear staining with Hoechst. Scale bar, 100µm on the left; 30µm on the right. **E.** The percentage of GFP-positive cells expressing specific maturation marker genes, representing keratinocyte maturation *in vivo*. n=6. Data are presented as the mean±SD of three independent experiments.

Keratinocyte gene expression *in vivo* is tightly controlled during embryogenesis 12. We examined how the global gene expression is temporally regulated on the *in vitro* platform. The cluster analysis of gene expression demonstrated that the global gene expression profiles clustered according to the differentiation stages (Figure 5A). A keratinocyte-enriched gene list was created based on published gene expression data 37(Supplemental Table S1). We observed that most keratinocyte-specific genes emerged when hESCs differentiate toward keratinocytes, and most gene expression reached maximal level around Day 11 and Day 26 (Figure 5B).

### Keratinocyte transplantation in mouse excisional wound model and maturation at air-liquid interface

To further evaluate the potential application of our hPSC-derived keratinocytes in clinical transplantation, we tested the *in vivo* survival and incorporation of hESC-derived keratinocytes in a mouse model. A GFP-labeled hESC reporter cell line was produced, and cells were differentiated to keratinocytes following the protocol above (Figure 4A). Cells were collected on day 20 and transplanted into excisional wounds on immunodeficient mice by injection and topical application 38. Seven days after transplantation, the wounds closed up (Figure 5C). H&E staining results confirmed formation of new cornified skin (Figure 5C). Immunostaining of serial skin sections showed positive expression of KRT14, KRT1, KRT10, IVL and FLG (Figure 5D, red fluorescence). GFP-positive cells remained in the skin, and the green fluorescence co-localized with the staining of keratinocyte maturation markers, indicating survival and continued maturation of the transplanted cells *in vivo* (Figure 5D-E). When grown at the air-liquid interface in 3D organotypic culture 29, hESC-derived keratinocytes further differentiated and matured two weeks after air lift, and some cells expressed maturation markers, such as KRT10 and IVL (Figure S5A).

## Discussion

Derivation of keratinocytes from pluripotent stem cells has many potential applications in disease modeling and clinical treatments. Combined with induced pluripotent stem cell (iPSC) technology, hPSCs have the potential to produce unlimited patient-specific keratinocytes *in vitro*. In order to realize the potential of hPSC-derived keratinocytes, new technologies need to be developed in many aspects, including lineage-specific differentiation, cell quality control, cell expansion and transplantation 39. In this report, we stagewisely established an *in vitro* epidermal differentiation procedure by the temporal manipulation of key signaling pathways. This procedure could generate >80% TP63-positive stage Ⅱ keratinocytes before the enrichment by keratinocyte culture conditions. This study not only provides an *in vitro* platform to understand human keratinocyte development, but also establishes a novel differentiation protocol to generate keratinocytes for potential therapeutic applications.

Our current knowledge on keratinocyte differentiation mainly comes from animal models, and the roles of various pathways in guiding the differentiation of keratinocytes from hPSCs have not been systematically studied. We showed that the interaction among TGFβ, BMP and WNT pathways are crucial for epidermal cell fate determination in cell culture. Consistent with *in vivo* studies 7, this report shows that TGFβ inhibition induces primitive ectoderm, and one-day TGFβ inhibition is sufficient for surface ectoderm induction. BMP4 activation and NOTCH inhibition is crucial to further increase epidermal gene expression (Figure S1A). The function of WNT signaling in ectodermal development is very critical as it regulates neural crest, neural plate border and epidermal cell fates in animal models, and this study highlighted that WNT signal is essential in the *in vitro* epidermal differentiation platform 4, 18. We also demonstrated an important function of WNT specific to hESC differentiation. Unlike mouse ESCs, hESCs can differentiate to extraembryonic lineages *in intro* 25. We demonstrated that WNT activation prevents extraembryonic lineage in epidermal differentiation, while all the other factors are reported extraembryonic inducers (Figure S1A) 25, 40. Taken together, we think that ectodermal sub-lineage cell fate is determined by the combinatorial effects of the above growth factors existing in differential gradients (Figure S5B).

Current protocols all use the low-calcium keratinocyte media to enrich keratinocytes, and differentiation efficiency is then analyzed using the enriched cells 41. Such approach could not accurately evaluate differentiation efficiency at earlier stages, because neural or neural crest cells generated earlier will be eradicated by low calcium conditions even when the purity of the starting population is low. Through temporal modulation of key signaling pathways, we are the first to generate >80% TP63-positive stage Ⅱ keratinocytes without enrichment. The global gene expression profiles demonstrate the sequential epidermal cell fate induction at each stage of differentiation (Figure 5). This procedure could serve as a valuable platform to understand keratinocyte cell fate determination. It is important to point out that other signaling pathways such as EGF, Sonic Hedgehog and retinoic acid are reported in epidermal differentiation. It would be interesting to see how modulators of those pathways could further improve the differentiation efficiency in the near future.

In this study, we also made a few interesting discoveries. First, TGFβ inhibition can be used to induce keratinocyte differentiation. Most previous protocols used retinoic acid to initiate epidermal lineage differentiation 19, 42, 43. However, retinoic acid is a strong neural lineage inducer and it leads to more neural differentiation compared to TGFβ inhibition-initiated procedure (Figure S5C). Second, we found that *KRT18* is not a suitable epidermal marker in E8-based differentiation platform. *KRT18* was previously reported to be a marker for surface ectoderm 13. We found that *KRT18* is already expressed on both mRNA and protein levels in undifferentiated hESCs cultured in E8 medium (Figure S5D and S5E). Therefore, we used *TP63*, but not *KRT18*, as the key marker to track the epidermal differentiation in this study. Third, we noticed that basal and suprabasal marker genes are dynamically expressed in our differentiation processes (Figure 4). Our results suggest that basal and suprabasal genes could be expressed in the same progenitor cells. It is supported by recent reports that basal and suprabasal genes can be co-expressed during specific stages of keratinocyte differentiation 44, 45. Fourth, we also revealed that calcium level is important for keratinocyte differentiation. A low calcium level is critical for the induction of *KRT14* in stage Ⅲ keratinocytes. However, the molecular mechanism is still to be explored.

In short, we developed a keratinocyte differentiation procedure in defined conditions, and rediscovered the essential roles of temporal and combinatory actions of TGFβ, BMP4, WNT and NOTCH signaling in keratinocyte differentiation at different developmental stages (Figure 1A). This differentiation procedure could serve as a valuable research platform for people to study epidermal differentiation *in vitro*. The protocol could also be used to generate patient-specific keratinocytes from iPSCs for cell therapy and disease models.

## Supplementary Material

Supplementary figures and tables.
