# Supplementary material for: Stagewise keratinocyte differentiation from human embryonic stem cells by defined signal transduction modulators
Source: Int J Biol Sci. 2020 Feb 21;16(8):1450–62. doi: 10.7150/ijbs.44414 (PMC7085224; doi:10.7150/ijbs.44414)
Supplement: Supplementary file 1 — Supplementary figures and tables. [file ijbsv16p1450s1.pdf]

Stagewise keratinocyte differentiation from human embryonic stem cells by defined signal transduction modulators

Hui Zhong[1], Zhili Ren[1], Xiaoyan Wang[1], Kai Miao[2], Wenjun Ni[3], Ya Meng[4], Ligong Lu[4,5], Chunming Wang[6], Weiwei Liu[1,7], Chu-Xia Deng[2], Ren-He Xu[1], Guokai Chen[1,7]

1. Centre of Reproduction, Development and Aging, Faculty of Health Sciences, University of Macau, Taipa, Macau
2. Cancer Centre, Faculty of Health Sciences, University of Macau, Taipa, Macau
3. Department of Urology Surgery, Zhuhai People's Hospital, Jinan University, Zhuhai, Guangdong 519000, China
4. Zhuhai Precision Medical Center, Zhuhai People's Hospital, Jinan University, Zhuhai, Guangdong 519000, China
5. Center of Interventional radiology, Zhuhai People's Hospital, Jinan University, Zhuhai, Guangdong 519000, China
6. State Key Laboratory of Quality Research in Chinese Medicine, Institute of Chinese Medical Sciences, University of Macau, Taipa, Macau
7. Bioimaging and Stem Cell Core Facility, Faculty of Health Sciences, University of Macau, Taipa, Macau

Correspondence: Guokai Chen, Ph.D., Faculty of Health Sciences, University of Macau, E12-4013, Taipa, Macau SAR, 999078. Phone: (853)-8822 4985; Fax: (853)-8822 2314; Email: guokaichen@um.edu.mo.

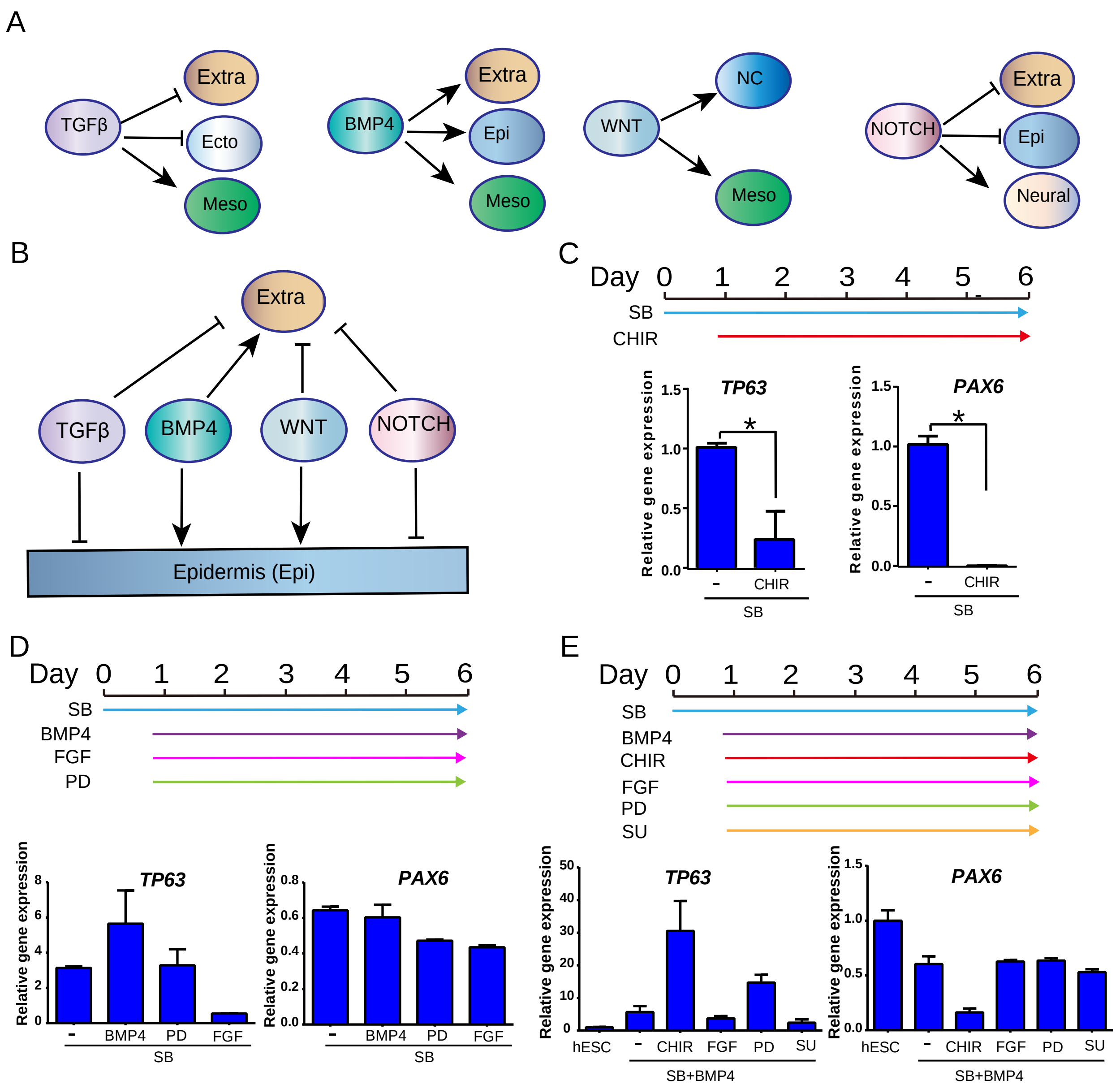

A
TGFβ
Extra
Ecto
Meso
BMP4
Extra
Epi
Meso
WNT
NC
Meso
NOTCH
Extra
Epi
Neural
B
Extra
TGFβ
BMP4
WNT
NOTCH
Epidermis (Epi)
C
Day 0 1 2 3 4 5 6
SB
CHIR
TP63
PAX6
Relative gene expression
*
-
CHIR
SB
D
Day 0 1 2 3 4 5 6
SB
BMP4
FGF
PD
TP63
PAX6
Relative gene expression
-
BMP4
PD
FGF
SB
E
Day 0 1 2 3 4 5 6
SB
BMP4
CHIR
FGF
PD
SU
TP63
PAX6
Relative gene expression
hESC
-
CHIR
FGF
PD
SU
SB+BMP4

Figure S1. Keratinocyte differentiation working model for stage I. Related to Figure 1.

A. Summary of current knowledge on signaling pathways involved in ectoderm differentiation[1-9]. Ecto, ectoderm. Meso, mesoderm. Epi, epidermis. Extra, extraembryonic. NC, neural crest.

B. Roles of key signaling pathways in keratinocyte differentiation based on our experimental results.

C. WNT activation helps suppress neural cell fate. hESCs were treated with SB431542 (SB) in differentiation medium, and CHIR99021(CHIR) was added from day 1 till day 6. *PAX6* expression was analyzed by RT-qPCR on day 6. The results were normalized to control without CHIR treatment; Data are presented as the mean±SD of three independent experiments. *, $p < 0.05$.

D. Effect of FGF pathway modulators on epidermal differentiation under SB431542 treatment. hESCs were treated with SB431542 (D0-6) in differentiation medium, and FGF2 (FGF), PD0325901 (PD) or BMP4 was added from day 1 to day 6. Cells were then harvested for gene expression analysis by RT-qPCR. The results were normalized to control without FGF modulators; Data are representative of three independent experiments.

E. Effect of FGF pathway modulators on epidermal differentiation under combined SB431542 and BMP4 treatment. hESCs were treated with SB431542 (D0-6) and BMP4 (D1-6) in differentiation medium, and FGF2 (FGF), PD0325901 (PD), SU-5402 (SU) or CHIR99021 (CHIR) was added from day 1 to day 6. Cells were then harvested for gene expression analysis by RT-qPCR. The results were normalized to control without FGF modulators; Data are representative of three independent experiments.

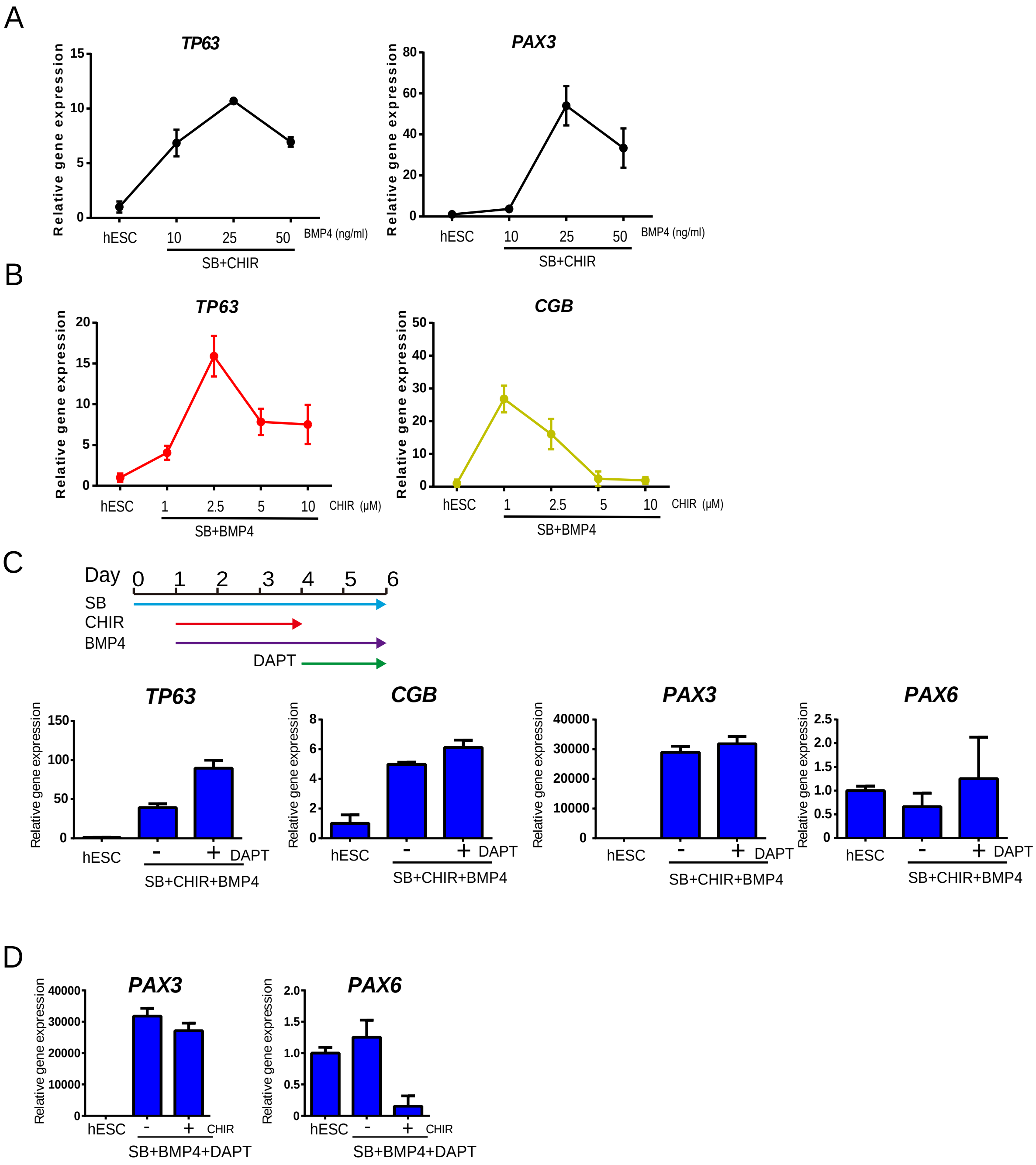

A
TP63
Relative gene expression
0
5
10
15
hESC
10
25
50
BMP4 (ng/ml)
SB+CHIR
PAX3
Relative gene expression
0
20
40
60
80
hESC
10
25
50
BMP4 (ng/ml)
SB+CHIR
B
TP63
Relative gene expression
0
5
10
15
20
hESC
1
2.5
5
10
CHIR (μM)
SB+BMP4
CGB
Relative gene expression
0
10
20
30
40
50
hESC
1
2.5
5
10
CHIR (μM)
SB+BMP4
C
Day
0
1
2
3
4
5
6
SB
CHIR
BMP4
DAPT
TP63
Relative gene expression
0
50
100
150
hESC
-
+
DAPT
SB+CHIR+BMP4
CGB
Relative gene expression
0
2
4
6
8
hESC
-
+
DAPT
SB+CHIR+BMP4
PAX3
Relative gene expression
0
10000
20000
30000
40000
hESC
-
+
DAPT
SB+CHIR+BMP4
PAX6
Relative gene expression
0
0.5
1.0
1.5
2.0
2.5
hESC
-
+
DAPT
SB+CHIR+BMP4
D
PAX3
Relative gene expression
0
10000
20000
30000
40000
hESC
-
+
CHIR
SB+BMP4+DAPT
PAX6
Relative gene expression
0
0.5
1.0
1.5
2.0
hESC
-
+
CHIR
SB+BMP4+DAPT

Figure S2. Optimization of epidermal cell fate determination at stage I. Related to Figure 2.

A. Effects of BMP4 dosage on epidermal differentiation. Cells were treated with increasing doses of BMP4 (D1-6) in the presence of SB431542 (SB, D0-6) and CHIR99021 (CHIR, D1-6). Samples were collected on day 6 for RT-qPCR. The results were normalized to hESC; Data are representative of three independent experiments.
B. Effects of CHIR99021 dosage on epidermal differentiation. Cells were treated with increasing doses of CHIR99021 (CHIR, D1-6) in the presence of SB431542 (SB, D0-6) and BMP4 (D1-6). Samples were collected on day 6 for RT-qPCR. The results were normalized to hESC; Data are representative of three independent experiments.
C. The impact of NOTCH inhibition (without concurrent CHIR99021 treatment) on epidermal cell fate determination. Cells were treated with SB431542 (SB, D0-6), BMP4 (D1-6) and CHIR99021 (CHIR, D1-3) with or without DAPT treatment from day 4 to day 6. Samples were collected on day 6 for RT-qPCR. The results were normalized to hESC; Data are representative of three independent experiments.
D. The impact of WNT activation along with NOTCH inhibition on neural and neural crest cell fate determination. CHIR99021 (CHIR) was added during DAPT treatment (Day 4-6), and the samples were collected on day 6 for RT-qPCR. The results were normalized to hESC; Data are representative of three independent experiments.

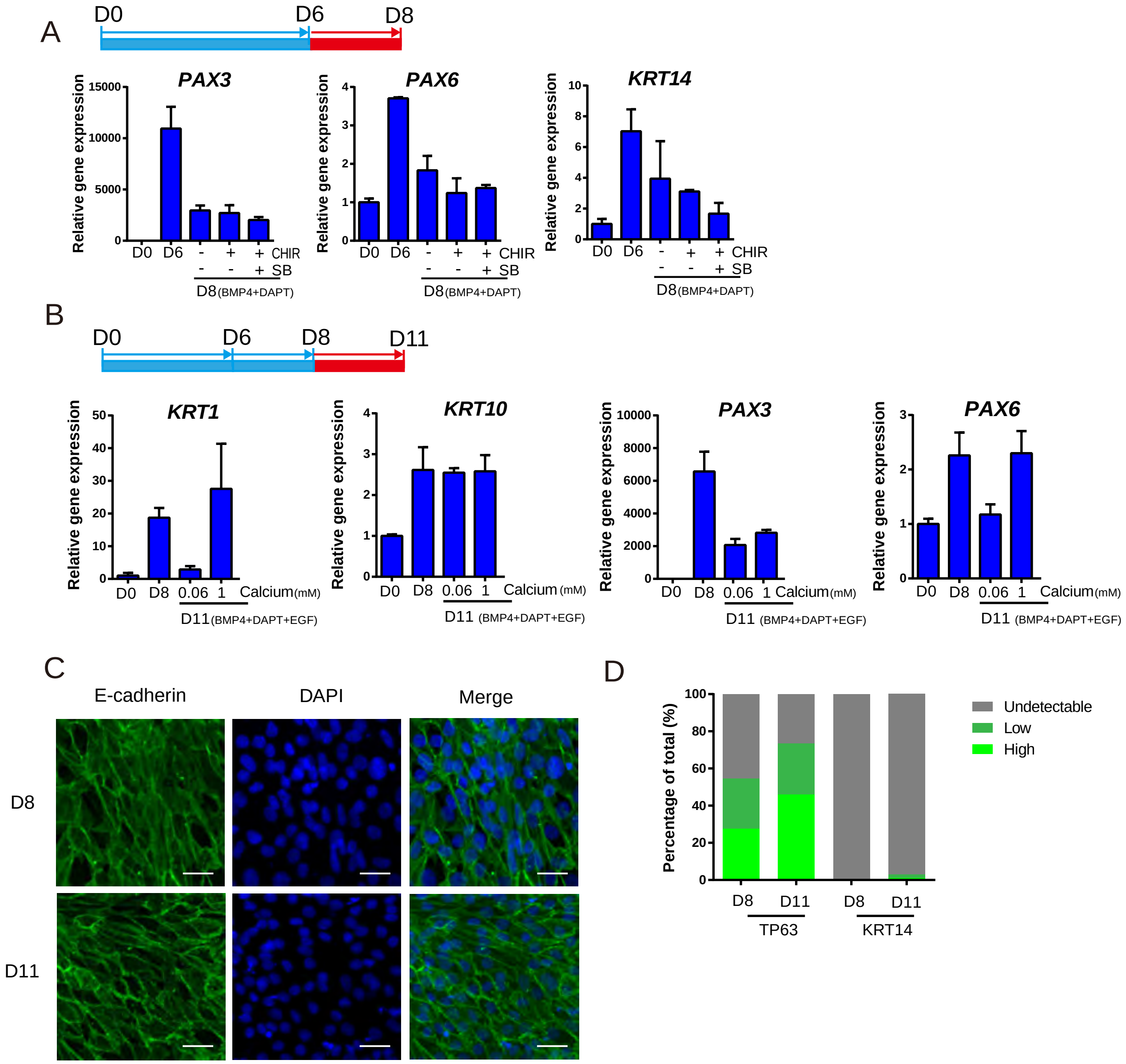

A
D0
D6
D8
PAX3
Relative gene expression
15000
10000
5000
0
D0 D6 - + + CHIR
- - + SB
D8(BMP4+DAPT)
PAX6
KRT14
B
D0
D6
D8
D11
KRT1
KRT10
PAX3
PAX6
D0 D8 0.06 1 Calcium(mM)
D11(BMP4+DAPT+EGF)
C
E-cadherin
DAPI
Merge
D8
D11
D
Percentage of total (%)
Undetectable
Low
High
D8 D11 D8 D11
TP63
KRT14

Figure S3. Optimization of conditions for epidermal keratinocyte formation in stage II and stage III. Related to Figure 3.

A. Continued TGFβ inhibition and WNT activation from day 6 to day 8 are not beneficial in epidermal differentiation. Beyond Day 6 of differentiation, cells were treated with or without SB431542 (SB) and CHIR99021 (CHIR) under BMP4 and NOTCH inhibitor treatment for two extra days, and the gene expression was examined by qPCR before and after the treatment. The results were normalized to hESC; Data are representative of three independent experiments.
B. The impact of calcium concentration on the expression of keratinocyte maturation markers and other lineage markers. After eight days of differentiation, cells were maintained in differentiation media with different calcium concentrations (1mM versus 0.06mM) from day 9 to day 11, and analyzed for gene expression before and after. The results were normalized to hESCs; Data are representative of three independent experiments.
C. Immunostaining of E-cadherin (green) expression on D8 and D11. Scale bar, 50 µm.
D. Quantification of Figure 3C by ImageJ. Gene expression levels are classified as high, low or undetectable.

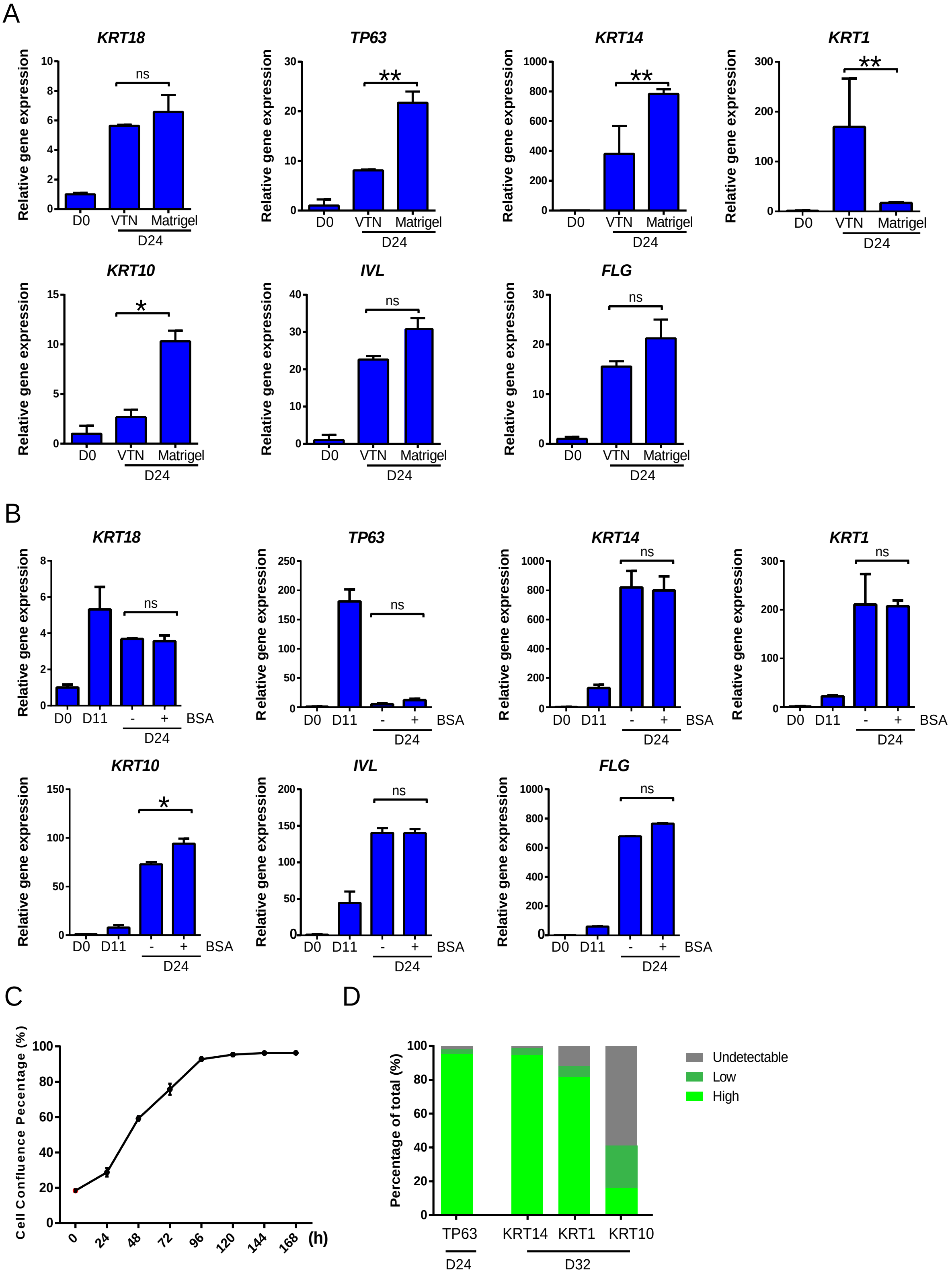

A
KRT18
TP63
KRT14
KRT1
KRT10
IVL
FLG
Relative gene expression
ns
**
*
D0
VTN
Matrigel
D24
B
KRT18
TP63
KRT14
KRT1
KRT10
IVL
FLG
Relative gene expression
ns
*
D0
D11
-
+
BSA
D24
C
Cell Confluence Pecentage (%)
0
24
48
72
96
120
144
168
(h)
D
Percentage of total (%)
Undetectable
Low
High
TP63
KRT14
KRT1
KRT10
D24
D32

Figure S4. Keratinocyte differentiation in defined system. Related to Figure 4.

A. Comparison of vitronectin (VTN) versus matrigel in keratinocyte differentiation. H1 cells were differentiated in VTN- or matrigel-coated plates. Samples were collected on day 0 and day 24. *KRT18, TP63*, *KRT14*, *KRT1, KRT10, IVN* and *FLG* expression was analyzed by qPCR. Results were normalized to undifferentiated hESCs. Data are presented as the mean±SD of three independent experiments. *, $p < 0.05$.

B. The impact of BSA on keratinocyte differentiation. Cells from day 11 of differentiation were further differentiated with or without BSA until day 24. Samples were collected on day 0, day 11 and day 24. *KRT18, TP63*, *KRT14*, *KRT1, KRT10, IVN* and *FLG* expression was analyzed by qPCR. Results were normalized to undifferentiated hESCs. Data are presented as the mean±SD of three independent experiments. *, $p < 0.05$.

C. Growth curve of hESC-derived keratinocytes monitored on IncuCyte for 168 hours (n = 12).

D. Quantification of Figure 4D by ImageJ. Gene expression levels are classified as high, low or undetectable.

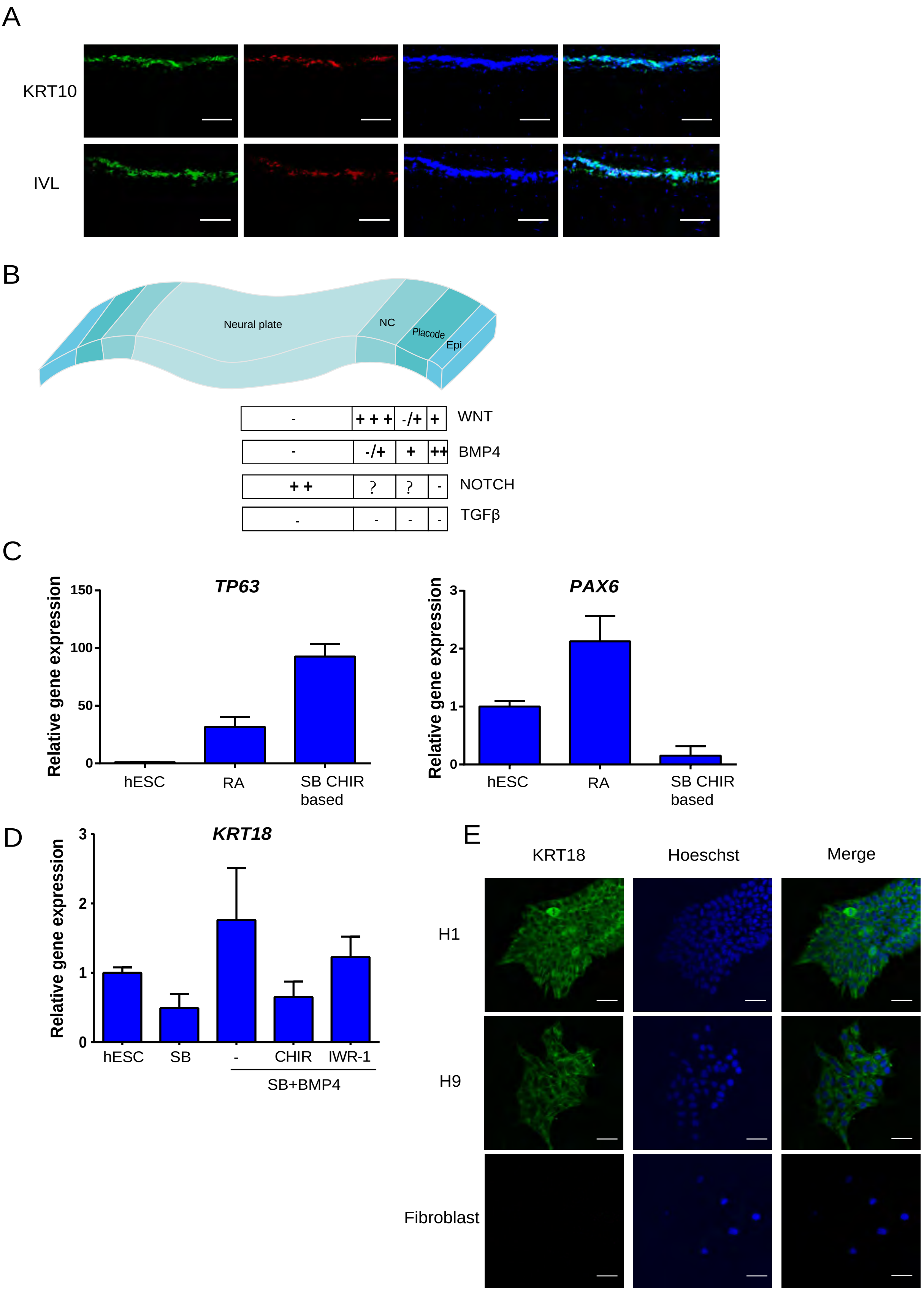
A
KRT10
IVL
B
Neural plate
NC
Placode
Epi
- | + + + | -/+ | + WNT
- | -/+ | + | ++ BMP4
+ + | ? | ? | - NOTCH
- | - | - | - TGFβ
C
TP63
Relative gene expression
150
100
50
0
hESC
RA
SB CHIR based
PAX6
Relative gene expression
3
2
1
0
hESC
RA
SB CHIR based
D
KRT18
Relative gene expression
3
2
1
0
hESC
SB
-
CHIR
IWR-1
SB+BMP4
E
KRT18
Hoeschst
Merge
H1
H9
Fibroblast

Figure S5. Roles of key signaling pathways in ectodermal and epidermal differentiation. Related to Figure 1 and Discussion.

A. Immunostaining of KRT10 and Involucrin (IVL) (red fluorescence) on serial sections of a 3D organotypic keratinocyte culture. Samples were collected 28 days after fibroblast coculture with keratinocytes derived from GFP-labeled hESCs (green fluorescence). Blue fluorescence, nuclear staining with Hoechst. Scale bar, 50µm.

B. Summary of the functions of key signaling pathways in cell fate determination in ectodermal tissues. NC, neural crest. Epi, epidermis. “-”, “+”, “++”, “+++” indicate the level of activity from low to high.

C. Comparison of retinoid acid-based (RA-based) differentiation method with TGFβ inhibition and WNT activation-based (SB/CHIR-based) method on keratinocyte induction. H1 cells were induced toward keratinocyte cell fate using either retinoid acid-based differentiation protocol (Retinoid acid (1µM) and BMP4 (10ng/ml) treatment for 6 days) or TGFβ inhibition and WNT activation-based differentiation protocol (SB431542 D0-6, CHIR99021 and BMP4 D1-6, DAPT D4-6). Samples were collected on day 6 and analyzed by RT-qPCR. The results were normalized to hESC; Data are representative of three independent experiments.

D. *KRT18* expression in hESCs versus differentiated cells. hESCs were treated with SB431542 (D0-6) with or without BMP4, CHIR99021 or IWR-1 (D1-6). *KRT18* expression was analyzed by RT-qPCR on day 6 and compared to the level in undifferentiated hESCs. The results were normalized to hESC; Data are presented as the mean±SD of three independent experiments.

E. Immunostaining of KRT18 in H1, H9 and fibroblasts. Scale bar, 50µm.

Supplemental Table S1. Skin-specific gene list generated according to BIOGPS

Table S1. Skin-specific gene list generated according to BIOGPS, Related to Figure 5								
Gene symbol	adult_kerat	H1_p35	Gene symbol	adult_kerat	H1_p35	Gene symbol	adult_kerat	H1_p35
HLA-G	8.7	7.1	ESRRAP2	9.0	7.2	RAPGEFL1	9.4	7.2
ZADH2	8.3	6.7	HLA-F	10.3	7.5	DSP	11.3	7.8
IL20RB	12.2	7.0	FAM129A	10.0	8.5	FCHSD2	8.8	6.8
OSGIN1	8.2	6.6	FERMT1	9.9	7.5	PRRG2	9.7	7.6
ITPRIP	10.7	8.9	CARD10	11.0	8.9	TMEM79	11.2	7.2
PARP14	9.1	7.1	LRP11	9.5	7.7	PPL	9.9	8.0
TIAF1	10.2	8.5	GFOD1	10.5	8.0	RAB25	11.2	8.6
UBALD2	8.9	7.2	LAD1	10.7	7.9	NUDT14	10.9	9.1
PCED1B	10.4	8.1	MICALCL	8.4	6.6	PROM2	10.6	6.9
SESN2	9.1	7.3	GCC1	10.1	8.4	TMEM54	10.4	7.4
NFIB	10.3	8.1	CDH3	13.2	10.5	CD55	9.5	7.9
SREBF1	9.0	7.4	CDS1	10.2	8.5	FAM83H	11.3	9.0
VIPR1	9.7	8.0	SLC20A2	10.9	8.5	INPP5D	10.1	8.0
MYO5A	9.6	7.9	IL1A	11.4	7.8	FAM160B2	8.6	6.9
PARP12	10.3	7.4	IRF6	10.4	7.2	MMP28	8.6	6.7
CYGB	8.9	7.3	S100A9	13.8	6.6	B4GALT1	9.3	7.0
EFHD2	10.4	8.6	CLDN1	12.4	8.0	JOSD1	9.4	7.8
EXOG	9.6	7.7	SLC9A1	11.7	9.2	NADSYN1	9.6	8.1
PCSK9	9.3	7.3	SCRIB	9.9	8.3	SDC4	11.4	8.0
MT2A	12.3	10.3	ZC3H12A	9.6	7.9	EVPL	10.2	7.2
SLC38A5	9.7	7.6	CITED4	11.3	7.5	CFLAR	10.4	7.2
TUBA4A	12.3	8.5	ADM2	9.8	8.2	IFITM3	13.3	11.1
MALL	10.7	7.6	MFSD6	8.7	7.1	PLAU	12.5	9.6
ARTN	8.8	6.8	NUPR1	11.4	7.2	ST6GALNAC2	9.2	6.6
THBS2	10.7	7.9	ISG20	9.1	7.5	CDH13	9.5	7.4
CDH4	8.8	7.0	RND3	11.7	9.5	ADAM8	9.4	6.9
FOXC1	11.9	7.1	RFTN1	10.2	8.5	WNT7A	9.3	6.7
CDA	10.4	7.0	TRIB3	12.5	7.8	CAMSAP2	11.1	8.6
MN1	9.1	7.3	IQGAP1	10.3	8.6	WNT10A	8.8	6.6
MOCOS	9.8	7.8	MMP9	11.9	7.6	TGM1	10.2	6.7
NFE2L1	11.6	10.1	MAP1LC3B	10.7	9.2	CCDC50	10.2	8.3
ZBTB43	9.6	7.8	FOXJ2	10.1	8.5	VAMP2	8.9	7.1
SARS	11.2	8.8	OLFML2A	9.7	7.3	NIPAL4	9.4	6.8
ULBP1	8.9	6.8	MYO1D	9.8	6.9	CXCL16	9.6	7.2
HERC6	9.8	8.1	UPP1	11.7	9.3	SPOCD1	9.5	6.7
ARHGEF4	8.7	6.7	CDK6	11.1	9.0	JUP	12.4	9.4
MX1	13.3	7.8	PMEPA1	9.9	7.9	IFIH1	8.8	6.6
FAT1	11.7	9.7	KCNK6	9.5	7.7	TAP1	11.5	9.5
IRX4	10.4	7.0	NBL1	10.2	8.5	ELL2	9.6	7.5
HOXC13	8.8	6.9	DST	9.9	6.9	LIPG	10.4	7.1
RNF39	9.0	7.1	PLCH2	12.3	6.9	HECA	8.5	7.0
HELZ2	10.6	8.9	HTRA1	11.8	8.6	GJB6	10.3	6.7
MTSS1	9.2	7.0	NUAK1	10.9	9.4	TINAGL1	8.3	6.7
ROBO3	9.2	7.4	HSD17B8	9.3	6.9	SQRDL	9.4	6.8
IRF7	9.6	7.3	SEMA3F	10.1	7.7	DHX32	10.1	7.9
TGFA	9.2	7.0	CNFN	10.5	9.0	KRT80	10.1	6.8
JAG2	9.2	7.3	PNPO	10.3	8.3	SPRR2D	11.2	6.8
PAK6	8.7	7.2	BST2	9.0	6.8	SBSN	10.7	6.7
ST14	9.9	7.9	EPB41L4A-AS	12.2	10.6	BHLHE40	11.7	8.7
PTPRK	11.2	8.9	JAG1	12.1	8.4	ELOVL1	9.0	7.4
RAB38	11.2	8.2	RTTN	10.7	9.1	CLIC3	11.2	6.9
BLCAP	10.5	8.2	STC2	11.4	9.3	ARHGDIB	9.9	7.5
HLA-B	12.4	8.6	NSMCE1	10.4	8.4	AFAP1L2	9.2	7.6
GALNT18	9.6	7.4	ZNF593	10.7	9.0	RPS6KA4	9.8	7.8
CELSR2	9.4	7.4	CASZ1	9.2	7.2	AXL	10.4	8.7
DLL1	8.7	7.1	MIF4GD	8.5	7.0	IGFBP3	9.1	7.5
NOTCH1	12.1	9.5	RIPK4	9.0	7.3	INPP1	9.6	7.9
TMEM51	10.8	8.8	SYK	8.5	7.0	COL17A1	13.1	6.9
IPO13	9.3	7.7	EIF1AY	8.4	6.7	PIK3IP1	9.9	6.7
OAS3	8.3	6.7	ASS1	11.0	8.4	CLEC16A	10.2	8.4
CDCP1	9.6	7.2	DPYSL4	10.7	9.2	RXRA	10.7	8.9
ITGA6	9.4	7.3	FEZ1	10.2	8.6	FTH1P12	12.2	10.7
STAT1	10.8	8.8	HES5	8.4	6.8	CLIP1	9.4	7.9
RHBDF2	9.2	7.5	HLA-A	13.2	11.3	PSTPIP2	8.9	7.1
SLC22A15	9.1	7.0	GSTO2	11.1	8.5	STAP2	8.5	6.9
DUSP5	11.0	9.3	LHPP	9.0	7.0	CEBPA	9.6	6.9
ETS2	10.7	9.1	LRP4	10.8	7.4	MET	8.4	6.8

Table S1. Skin-specific gene list generated according to BIOGPS, Related to Figure 5								
Gene symbol	adult_kerat	H1_p35	Gene symbol	adult_kerat	H1_p35	Gene symbol	adult_kerat	H1_p35
PERP	11.3	8.3	ZNF750	10.2	6.6	IER3	12.5	9.4
ITGB4	10.8	6.9	NFIX	9.5	6.7	TRAF3IP2	9.8	7.7
LYPD3	10.9	6.7	PVR	9.7	7.2	ADRB2	10.5	7.4
TACSTD2	14.0	7.3	IFI35	9.8	7.1	UAP1	10.3	7.7
EFNA1	10.3	7.1	WDR66	9.3	6.9	GBP1	8.3	6.8
RAB5B	12.0	10.5	DENND2C	8.8	7.2	PYGL	10.9	8.6
PLCD1	9.5	7.2	CDC25B	9.8	7.9	FZD6	9.0	7.4
PPP1R18	10.7	8.8	TSPAN1	9.1	7.1	LIMA1	11.4	9.2
B2M	13.1	9.3	CA12	9.0	7.1	PTGFRN	10.5	7.9
DUSP14	11.6	8.4	KRT5	14.3	6.8	ASAP2	9.2	7.3
GPR87	8.7	6.8	SLC13A5	9.8	7.0	IGSF3	10.0	8.3
BTG1	11.9	10.0	KRT6A	14.2	6.8	DGKA	9.3	7.5
TNFAIP1	9.9	7.9	LAMA3	11.8	6.9	CLTB	12.5	9.0
PRSS12	8.9	7.2	SDC1	11.0	7.6	JUNB	10.0	6.8
EPSTI1	10.5	7.7	LAMB3	12.5	6.7	MYH9	13.7	12.1
SEMA3C	8.9	6.7	TTPAL	9.7	8.2	SH3KBP1	10.0	8.4
NDE1	11.1	8.0	HSF2BP	8.5	6.8	CSRP1	12.6	10.8
DLK2	8.6	6.8	KRT6B	13.3	6.8	CD151	12.4	10.5
DOCK6	9.1	7.3	BAG3	10.3	8.7	CYB5R1	11.9	7.8
WASF2	10.3	8.6	KRT14	14.7	6.7	ACKR3	9.6	7.5
LGALSL	9.9	7.9	AVPI1	11.9	8.8	TIMP3	9.3	7.0
LEMD1	8.2	6.6	SERPINB5	12.8	7.2	CPA4	12.0	6.9
RGS12	8.8	7.0	DECR1	10.9	9.1	PPP1R14C	10.6	6.7
FAM110A	9.0	7.2	RAC2	10.1	7.1	MICALL1	10.4	8.2
P2RX4	8.6	7.0	COPZ2	8.5	6.7	TAX1BP3	12.5	10.7
CBLC	9.9	7.1	TSC22D3	8.9	6.9	RAB11FIP5	9.6	7.5
FBXW4	10.1	8.1	CYBRD1	9.4	7.4	S100A3	9.8	6.6
FGF11	9.7	7.3	PYGB	11.1	8.3	GIPC1	11.6	9.4
ZNF385A	9.2	6.8	PHLDA3	10.3	8.7	PLEK2	10.2	6.7
XAF1	9.2	6.6	PLD1	8.4	6.8	PTGS2	11.3	7.2
IFI44L	9.5	6.5	CEBPB	12.8	10.4	SERPINB2	10.4	6.8
MKNK2	11.4	9.8	TEX2	9.8	7.7	TBC1D2	10.2	7.4
TEF	8.7	6.8	GADD45B	9.5	7.5	KLF4	8.5	6.9
PPP1R3C	8.6	6.4	UBASH3B	9.1	7.3	RHOD	12.8	7.9
IRX3	10.9	7.4	HIST2H2AC	11.2	8.0	TNFRSF12A	11.2	9.7
ZNF467	8.2	6.7	EXT1	10.2	8.5	PTRF	12.6	10.3
H1FX	11.3	9.3	HSPB1	14.7	11.0	SPRYD3	9.5	7.1
STK3	10.3	8.3	TOB1	9.9	8.0	TAPBP	10.1	7.5
RILPL1	10.1	7.7	BMP1	8.6	6.8	ITGA3	11.8	8.3
TPD52L1	8.7	7.1	GALNT6	9.3	6.8	THBS1	11.9	8.6
HOXA5	8.7	7.0	KRT16	13.8	6.8	COBLL1	10.4	8.6
HOXA9	8.9	6.6	EHD2	10.1	6.8	ENDOD1	9.2	6.8
PKD1	9.3	7.4	NT5E	9.3	7.0	CAPG	9.0	6.9
PPM1F	9.9	8.4	CD44	10.5	7.8	EDEM1	8.9	7.2
ZP3	8.9	7.0	DKK3	11.4	8.5	ANKRD13A	10.2	8.1
GAA	8.8	7.2	SPSB1	9.1	6.8	HES2	10.9	6.8
USP3	9.2	7.3	PLP2	9.0	7.0	ACTN4	12.5	10.5
LAMP3	8.9	6.7	ZBED2	9.9	6.7	CASP4	9.1	6.8
PEX2	10.2	8.4	DPP9	10.5	9.0	LAMC2	12.8	7.8
ISG15	12.6	9.3	GPX3	10.7	8.3	TNFAIP3	9.4	7.2
STOM	11.0	8.0	LDLR	12.3	10.7	CSRNP1	9.7	8.1
BNIPL	9.0	6.9	TMEM173	8.9	6.6	KRT7	11.4	6.9
HS1BP3	8.8	6.6	SLC31A2	10.7	7.1	NFKB1	11.1	8.6
H1F0	12.6	8.7	ARHGDIA	11.8	9.6	IGFBP7	11.7	8.1
TINF2	10.3	8.0	MYLK	8.4	6.8	TIPARP	10.6	7.7
SAT1	12.9	8.9	COMT	10.5	8.0	GJB2	12.2	7.4
SYBU	8.7	6.8	LARP6	10.3	8.2	ITGA2	10.6	7.5
BCL2L2	9.8	7.6	PPP1R13B	9.0	7.5	ALDH3B2	9.5	6.7
EPHB3	8.7	6.8	QSOX1	9.8	7.7	GOLGB1	10.0	7.9
HES4	11.8	8.6	FOXD1	9.4	7.4	KCTD11	8.8	7.1
KIAA0556	8.7	7.2	PSMB8	8.8	7.1	TM4SF1	11.5	7.1
CXCL14	10.3	6.9	FXYD5	11.5	9.3	HACD2	11.4	9.4
RALGDS	9.4	7.9	PHLDB2	9.0	7.0	ATF6	9.8	8.2
CHRNB1	9.9	7.7	PRNP	12.6	10.3	ZNF185	10.6	7.4
IER5L	8.7	6.7	HBEGF	10.2	7.9	ANXA8	12.2	6.9
TP63	11.4	6.8	ACSL1	8.7	6.9	NDST1	8.8	7.2
FGFBP1	12.6	6.7	PGF	8.7	6.7	CYB561A3	10.3	8.1

Table S1. Skin-specific gene list generated according to BIOGPS, Related to Figure 5

Gene symbol	adult_kerat	H1_p35	Gene symbol	adult_kerat	H1_p35	Gene symbol	adult_kerat	H1_p35
EDN1	11.6	9.0	SIRPA	10.2	7.3	MGST2	10.5	8.9
CMIP	10.1	8.3	RRAGC	10.2	8.2	ZDHHC1	8.4	6.7
MNT	9.8	7.9	MGC72080	8.7	7.1	TRPM4	10.0	7.4
S100A13	9.2	7.5	ANXA8L1	9.7	6.9	PAQR7	9.2	7.2
BCAR3	9.9	8.3	TMEM141	9.9	8.2	CAMK2N1	11.7	8.5
KANK4	10.4	6.6	UBAP1	10.5	8.9	VGLL1	8.6	6.6
CRYAB	8.6	6.8	ADAM15	10.8	8.1	COL7A1	13.4	11.4
LCOR	9.0	7.5	DUSP1	10.4	8.2	PPP1R13L	11.2	7.2
SERINC2	10.3	7.3	PTHLH	10.1	6.8	TSPAN31	9.8	8.3
CHURC1	10.2	7.4	CD2BP2	11.5	9.9	AKR1C2	8.7	6.7
CTIF	9.5	7.2	SERTAD1	10.8	8.5	TFAP2A	10.6	6.9
SPHK1	9.7	6.8	ARSA	8.4	6.8	BTG2	9.8	7.3
TNFRSF6B	8.7	6.8	DSG3	12.3	6.6	SERTAD2	10.3	7.9
SH3PXD2A	11.8	7.2	NUDT18	8.5	6.8	SPRR1A	13.1	6.6
TMEM154	10.8	6.8	MAFB	8.9	7.1	SPRR1B	12.7	6.7
GBP2	9.1	7.0	VEGFC	9.5	6.7	CACNB3	9.6	7.7
S100P	8.7	6.6	SLC50A1	9.6	8.0	PIGQ	9.4	7.8
ELMO3	9.6	7.4	MMP10	10.8	7.0	DHRS1	11.6	8.2
RGS10	9.3	7.5	SAMD9	9.1	6.9	MAP3K6	9.2	7.4
KRT17	14.0	7.0	HIST1H1C	11.0	7.7	NENF	11.4	9.4
S100A6	13.8	9.3	CD99L2	9.6	7.6	TRIM29	9.7	6.8
FAM89B	9.6	7.7	LGALS1	13.5	9.4	SH3RF2	10.7	7.0
MFAP5	10.0	7.2	MSLN	10.6	6.9	MAN2B2	9.8	6.8
COL16A1	10.9	7.2	CD82	8.3	6.8	STARD10	11.5	7.6
LMNA	11.2	8.8	TMEM45A	9.6	7.9	TYMP	12.9	7.0
ST3GAL5	9.2	7.1	LTB4R	9.3	6.8	SH3BGRL3	12.4	9.0
ELF4	9.4	7.3	HIST1H2BD	10.0	7.6	PRDM4	9.5	8.0
FST	13.0	9.4	S100A8	11.3	6.9	STIM1	9.2	7.2
ATP2B4	9.6	7.4	RNH1	10.1	8.0	DFNA5	11.6	8.7
GPC1	10.0	7.0	ATP6V0D1	10.7	9.0	IER5	9.8	7.6
FAM46A	10.4	8.4	HIST1H2BK	11.0	8.8	RNF149	9.9	8.3
H19	10.8	6.8	ANTXR2	10.2	7.5	MPZL2	9.9	6.8
ARL8B	10.5	8.7	BAIAP2	8.7	7.0	TUFT1	12.4	8.4
HERPUD1	10.2	8.5	FSTL3	11.4	7.1	EGR1	10.1	7.5
MVP	9.5	6.7	RAB7B	8.9	6.8	NCOA7	9.4	7.6
RHOC	12.1	9.5	SPATA20	9.2	7.7	RAB32	11.4	8.7
MXRA5	10.0	7.4	MSN	12.0	10.4	P4HA2	9.6	7.7
OBFC1	9.7	7.7	LPXN	9.2	7.2	CDKN1A	13.3	9.4
KHNYN	9.6	7.5	SNAI2	9.4	6.9	TGFBI	13.6	7.6
SUN2	11.3	8.8	RRAS	12.0	9.2	CST3	12.8	9.9
LTBR	9.8	7.1	VAMP3	11.3	9.1	EMP1	10.2	6.8
MGAT1	10.6	9.1	RIN1	8.6	7.0	MBOAT7	10.2	8.2
SDSL	8.4	6.8	ABCC3	9.6	6.7	PEX11B	10.0	8.2
ECM1	9.2	6.9	RETSAT	8.7	7.2	CD63	10.4	8.7
GRN	11.6	9.2	UBXN6	9.9	8.0	CERCAM	9.5	6.8
IL10RB	9.2	7.4	IL13RA1	10.2	8.1	LY6E	12.2	9.9
GSTO1	11.6	9.9	TICAM1	10.0	6.8	ARID5B	9.8	7.3
IAH1	9.7	8.2	TMEM106C	10.0	8.4	DAZAP2	11.6	9.3
PHF11	9.9	8.1	ACOX3	9.2	7.5	BLVRB	9.7	8.1
TMBIM1	10.3	7.6	RSPH3	8.6	6.8	DNASE2	10.6	9.0
ZFP36	10.6	8.7	DSC2	10.5	8.2	IFI16	10.1	6.7
KLF6	12.0	8.4	ECHDC2	10.7	8.5	NDRG1	11.6	7.1
SERPINB7	11.1	6.8	ANXA3	9.9	7.6	HIST2H2AA3	11.3	7.5
SIK1	9.9	6.8	SFN	14.0	7.0	STARD5	8.6	6.9
FAM129B	10.7	8.9	TCP11L1	8.8	7.1	S100A11	12.6	9.5
KCTD10	9.4	7.7	VAMP8	11.6	10.0	GSTK1	11.2	9.0
ASB1	9.6	8.0	CDC42EP5	10.6	6.8	PLSCR3	12.5	11.0
MYO1B	10.1	8.1	TRIM4	9.1	6.8	ANXA2P1	12.6	10.7
CBX4	10.2	6.9	FAM89A	8.9	7.0	S100A16	12.9	7.7
GPR108	9.0	7.3	CEBPD	12.3	7.3	AHNAK	11.0	7.8
KLHDC8B	10.3	8.1	PPP1R15A	12.0	9.2	FILIP1L	9.9	6.9
HIST2H2BE	8.9	7.1	HOMER3	9.9	7.4	EPAS1	10.6	8.0
OAF	9.1	6.8	GUK1	12.3	9.5	OPTN	9.5	7.0
PQLC1	11.3	9.4	HLA-E	10.3	7.1	CTSB	11.5	8.7
TOLLIP	8.9	7.2	CCDC92	10.9	6.9	PALLD	12.2	10.3
MXD1	8.6	6.8	DHRS7	10.8	9.3	FAM214B	9.4	7.4
DEFB1	10.1	6.6	ZNF518B	8.6	7.1	MOB3A	8.6	7.0

Table S1. Skin-specific gene list generated according to BIOGPS, Related to Figure 5

Gene symbol	adult_kerat	H1_p35	Gene symbol	adult_kerat	H1_p35	Gene symbol	adult_kerat	H1_p35
DYRK4	10.0	7.8	IFITM2	13.2	11.2	VPS28	10.4	8.2
SIL1	9.7	7.1	AGRN	10.7	8.8	CCNO	8.4	6.7
FKBP9P1	10.4	7.1	ARRDC4	8.6	6.9	CUL9	9.5	6.7
ZFHX3	9.3	7.8	KRTDAP	12.9	6.7	IFI6	13.5	7.7
KRCC1	8.8	7.1	PLXNA3	9.2	7.3	SOX9	8.8	7.1
SYTL1	9.5	7.1	PPP2R5B	8.7	6.9	LAMB2	9.7	8.0
GSN	8.7	6.7	SLC16A3	11.0	7.5	LGALS3BP	10.5	7.7
PRSS23	10.5	7.8	TXNIP	12.2	6.9	KIFC2	9.1	7.3
STX12	9.0	7.4	CHP1	9.9	8.0	NAPRT	10.7	9.0
ZBTB4	10.3	8.2	MGMT	10.5	8.2	TNFRSF14	9.9	6.7
KLHL22	9.1	7.0	IFI27L2	10.5	7.2	TMEM63B	9.4	7.0
THBS3	8.9	6.6	POMGNT1	10.6	8.6	ZNF524	9.7	7.6
MSRB2	11.5	9.3	HOXC8	8.4	6.8	FAM57A	10.5	8.9
PMM1	10.5	8.5	SIDT2	10.0	8.4	RAD23A	11.1	9.0
ITGA5	10.4	8.1	DNAJB2	11.4	8.6	GPNMB	8.7	6.9
MAP7D1	12.4	10.0	TPP1	10.1	8.5	LPAR5	9.7	6.8
MAOA	10.6	7.0	ZFAND2B	9.2	7.7	NDUFA4L2	8.6	6.8
RHOG	11.1	9.0	RHBDD2	10.8	9.0	ATG7	9.7	8.2
IRAK1	11.0	8.8	TMEM134	10.3	8.7	ERCC1	10.2	8.2
CHMP3	8.9	7.1	GADD45A	11.8	9.6	ACSS2	10.1	7.3
FLRT2	9.1	6.9	LYPD6B	8.5	6.9	GSS	9.8	8.2
MAPKBP1	8.4	6.9	DDIT4	13.0	8.7	SPOP	10.3	8.7
MBD6	9.9	8.2	ZNF503	9.9	6.6	BCYRN1	11.3	9.5
DUSP18	8.9	7.3	TP53TG1	8.7	7.1	D2HGDH	9.7	7.5
SLC44A2	11.7	9.8	WLS	8.6	6.9	LGALS7	13.7	6.7
TNC	10.8	7.7	DLX5	8.5	6.8	CLSTN1	11.5	9.5
INPP4B	9.4	7.0	S100A14	10.8	6.5	SPINK5	8.3	6.8
MBNL2	9.0	6.9	RBCK1	10.6	7.9	RAB3GAP1	11.6	9.8
MYOF	10.7	8.4	TCF25	11.2	9.6	RABGAP1	10.9	9.2
ADGRG1	12.0	6.9	LRRC41	9.3	7.8	EFNB1	9.6	6.6
STAT2	11.2	9.3	EVI5L	10.0	8.1	SMTN	9.1	7.1
GAS6	11.2	7.8	COMMD3	10.7	7.8			
IRF9	10.9	8.4	WDR13	10.4	8.0			
TMEM175	9.1	7.6	PRR14	10.5	8.6			
SLC25A28	10.4	8.7	FLRT3	9.9	7.3			
VPS37B	10.2	8.0	BGN	9.8	7.4			

Supplemental Table S2. Primer list

Primer	Sequence
TP63-F*	GGAAAACAATGCCCAGACTC
TP63-R*	GTGGAATACGTCCAGGTGGC
KRT14-F	GACCATTGAGGACCTGAGGA
KRT14-R	ATTGATGTCGGCTTCCACAC
KRT1-F	AGAAAGCAGGATGTCTGG
KRT1-R	AAACAAACTTCACGCTGG
KRT10-F	ACGAGGAGGAAATGAAAGAC
KRT10-R	GGACTGTAGTTCTATCTCCAG
IVL-F	TCCTCCAGTCAATACCCATCAG
IVL-R	CAGCAGTCATGTGCTTTTCCT
FLG-F	TGAAGCCTATGACACCACTGA
FLG-R	TCCCCTACGCTTTCTTGTCCT
CGB-F**	TCACCGTCAACACCACCATC
CGB-R**	AGAGTGCACATTGACAGCTG
PAX3-F	AATTACTCAAGGACGCGGTC
PAX3-R	TTCTTCTCGCTTTCCTCTGC
PAX6-F	CACCTACAGCGCTCTGCCGC
PAX6-R	CCCGAGGTGCCCATTGGCTG
POU5F1(OCT4)-F	AACCTGGAGTTTGTGCCAGGGTTT
POU5F1(OCT4)-R	TGAACTTCACCTTCCCTCCAACCA
NANOG-F	GATGCCTCACACGGAGACTG
NANOG-R	GCAGAAGTGGGTTGTTTGCC
GAPDH-F	GTGGACCTGACCTGCCGTCT
GAPDH-R	GGAGGAGTGGGTGTCGCTGT
TBP-F	TGAGTTGCTCATACCGTGCTGCTA
TBP-R	CCCTCAAACCAACTTGTCAACAGC

*TP63 primers detect the *ΔNp63* isoform [10].

**CGB primers detect a conserved sequence that is shared by *CGB2, CGB3, CGB7* and *CGB8* [11].

Supplemental Reference

1. Baonza A, Freeman M. Notch signalling and the initiation of neural development in the Drosophila eye. Development. 2001; 128: 3889-98.
2. Chambers SM, Fasano CA, Papapetrou EP, Tomishima M, Sadelain M, Studer L. Highly efficient neural conversion of human ES and iPS cells by dual inhibition of SMAD signaling. Nature biotechnology. 2009; 27: 275-80.
3. Haider S, Meinhardt G, Saleh L, Fiala C, Pollheimer J, Knofler M. Notch1 controls development of the extravillous trophoblast lineage in the human placenta. Proceedings of the National Academy of Sciences of the United States of America. 2016; 113: E7710-E9.
4. Gadue P, Huber TL, Paddison PJ, Keller GM. Wnt and TGF-beta signaling are required for the induction of an in vitro model of primitive streak formation using embryonic stem cells. Proceedings of the National Academy of Sciences of the United States of America. 2006; 103: 16806-11.
5. Laura Menendeza TAY, Parker B. Antinb, and Stephen Dalton. Wnt signaling and a Smad pathway blockade direct the differentiation of human pluripotent stem cells to multipotent neural crest cells. Proceedings of the National Academy of Sciences. 2012; 109: 9220-.
6. Roberts RM, Loh KM, Amita M, Bernardo AS, Adachi K, Alexenko AP, et al. Differentiation of trophoblast cells from human embryonic stem cells: to be or not to be? Reproduction. 2014; 147: D1-12.
7. Wilson PA, Hemmati-Brivanlou A. Induction of epidermis and inhibition of neural fate by Bmp-4. Nature. 1995; 376: 331-3.
8. Xu RH, Chen X, Li DS, Li R, Addicks GC, Glennon C, et al. BMP4 initiates human embryonic stem cell differentiation to trophoblast. Nature biotechnology. 2002; 20: 1261-4.
9. Yu P, Pan G, Yu J, Thomson JA. FGF2 sustains NANOG and switches the outcome of BMP4-induced human embryonic stem cell differentiation. Cell stem cell. 2011; 8: 326-34.
10. Kidwai FK, Liu H, Toh WS, Fu X, Jokhun DS, Movahednia MM, et al. Differentiation of human embryonic stem cells into clinically amenable keratinocytes in an autogenic environment. The Journal of investigative dermatology. 2013; 133: 618-28.
11. Weber S, Eckert D, Nettersheim D, Gillis AJ, Schafer S, Kuckenberg P, et al. Critical function of AP-2 gamma/TCFAP2C in mouse embryonic germ cell maintenance. Biol Reprod. 2010; 82: 214-23.
